# Supplementary material for: Tertiary Structures of Haseki Tick Virus Nonstructural Proteins Are Similar to Those of Orthoflaviviruses
Source: Int J Mol Sci. 2024 Dec 20;25(24):13654. doi: 10.3390/ijms252413654 (PMC11678601; doi:10.3390/ijms252413654)
Supplement: Supplementary file 1 [file ijms-25-13654-s001.zip › Supplementary material_rev.pdf]

Table S1. Putative HSTV nonstructural transmembrane proteins.

Protein name	Transmembrane domain name	HSTV polyprotein coordinates	Size of transmembrane domain, a.a.
NSTR1	NSTR1-D1	1272-1296	21
	NSTR1-D2	1303-1320	17
	NSTR1-D3	1345-1369	24
	NSTR1-D4	1405-1429	24
NSTR2	NSTR2-D1	2833-2855	23
	NSTR2-D2	2875-2900	26
	NSTR2-D3	2921-2938	18
	NSTR2-D4	2959-2978	20
C-terminal transmembrane protein	Transmembrane region 1	4910-4929	20
	Transmembrane region 2	4942-4964	23
	Transmembrane region 3	5045-5078	34
	Transmembrane region 4	5085-5104	20

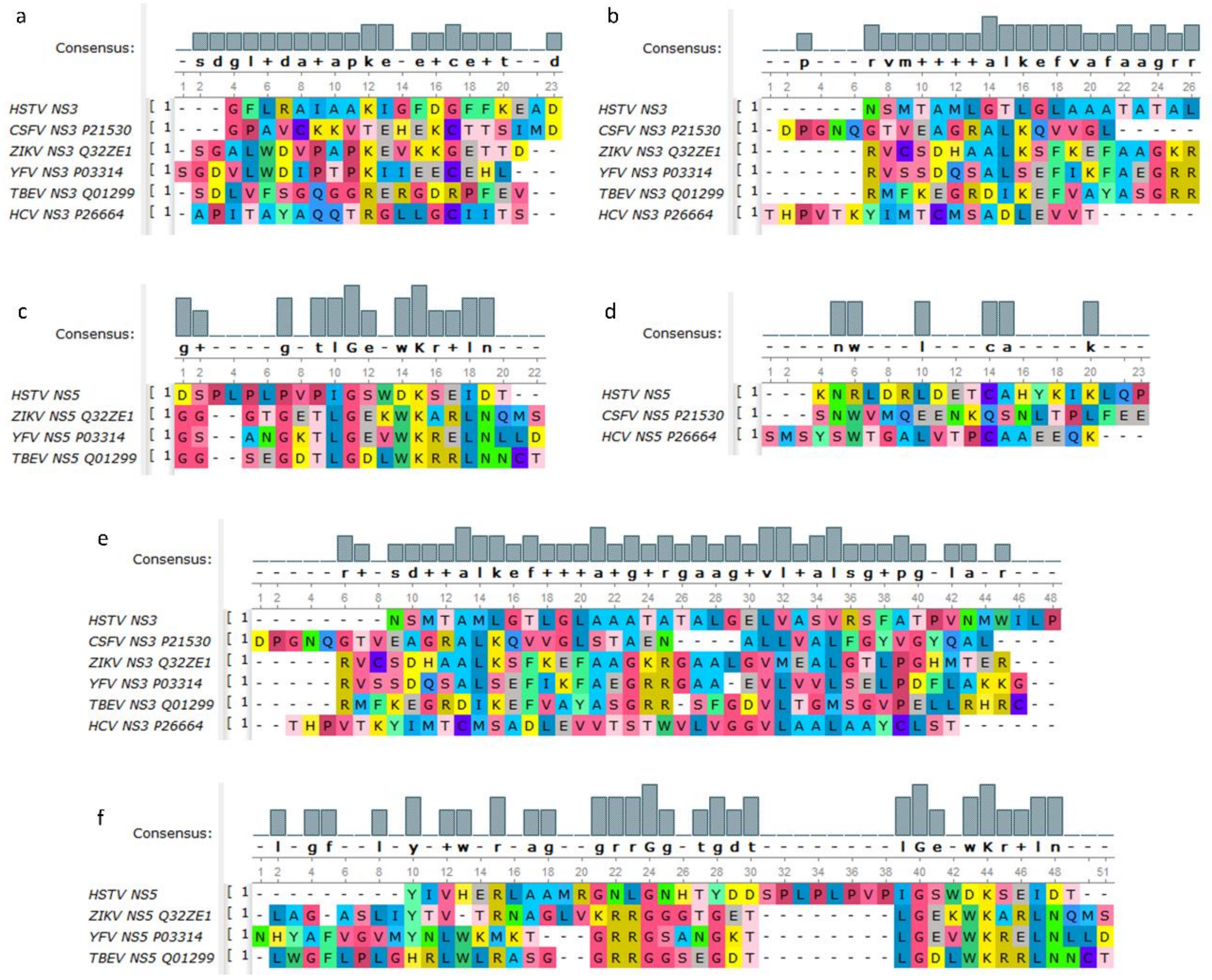

Figure S1. The amino acid sequence alignment of N-terminus and C-terminus of HSTV NS proteins: (a) the N-terminus of HSTV NS3 was aligned with the N-termini of NS3: CSFV (UniProtKB accession number: P21530), ZIKV (UniProtKB accession number: Q32ZE1), YFV (UniProtKB accession number: P03314), TBEV (UniProtKB accession number: Q01299), HCV (UniProtKB accession number: P26664); (b) the C-terminus of HSTV NS3 was aligned with the C-termini of NS3: CSFV (UniProtKB accession number: P21530), ZIKV (UniProtKB accession number: Q32ZE1), YFV (UniProtKB accession number: P03314), TBEV (UniProt: Q01299), HCV (UniProt: P26664); (c) the N-terminus of HSTV NS5Mtase was aligned with the N-termini of NS5: ZIKV (UniProtKB accession number: Q32ZE1), YFV (UniProtKB accession number: P03314), TBEV (UniProtKB accession number: Q01299); (d) the N-terminus of HSTV NS5RdRp was aligned with the N-termini of NS5b (RdRp): CSFV (UniProtKB accession number: P21530), HCV (UniProtKB accession number: P26664); (e) the C-terminus of HSTV NS3 and the N-terminus of HSTV NSTR2 (putative NS4A analogue) was aligned with the C-termini of NS3 and the N-termini of NS4A: CSFV (UniProtKB accession number: P21530), ZIKV (UniProtKB accession number: Q32ZE1), YFV (UniProtKB accession number: P03314), TBEV (UniProtKB accession number: Q01299), HCV (UniProtKB accession number: P26664); (f) the C-terminus of HSTV NSTR2 (putative NS4B analogue) and the N-terminus of HSTV NS5Mtase was aligned with the C-termini of NS4B and the N-termini of NS5: ZIKV (UniProtKB accession number: Q32ZE1), YFV (UniProtKB accession number: P03314), TBEV (UniProtKB accession number: Q01299)

Table S2. Putative cleavage sites of HSTV NS3pro.

	**Polyprotein coordinates of putative NS3pro HSTV proteolysis sites**	**Amino acids sequence of putative NS3pro HSTV proteolysis sites**	**HSTV polyprotein proteolysis region**
**Ortoflavi-like cleavage site**	442-444	**KR↓G**	
	450-452	**RR↓S**	
	604-606	**RR↓A**	
	705-713	HVI**KR↓G**DTG	
	1582-1590	FTT**RK↓S**APY	NS2B↓NS3pro
	2055-2057	**RK↓A**	
	2109-211	**RR↓G**	
	3424-3432	ECR**KR↓A**AAA	NS4B↓NS5MT
	3640-3642	**RR↓G**	
	3737-3739	PAS**KR↓A**GRC	NS5MT↓NS5A-NS5RdRp
	3908-3910	**RK↓G**	
	4619-4621	**RK↓S**	
	4785-4787	SEG**RR↓G**PRD	
	4885-4887	**RR↓A**	
	4930-4932	**RK↓S**	
	5005-5007	**RR↓G**	

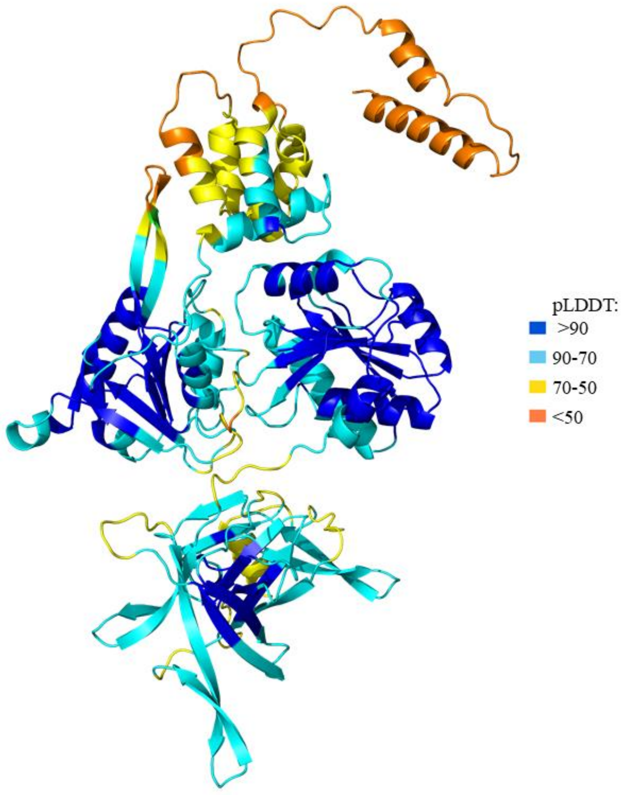

Figure S2. Tertiary structure model of HSTV NS3 in pLDDT color.

Table S3. Comparison of amino acid sequences and tertiary structure of HSTV NS3 with NS3 of *Flaviviridae* family viruses.

**NS3 PDB ID**	**Name of virus**	**TM-score**	**RMSD, Å**	**Aligned residues, a.a.**	**Amino acid sequence identity, %**
2VBC	Dengue virus 4	0.6	5.43	337	13
5FPT	Hepatitis C virus	0.47	3.88	306	19
5WX1	Classical swine fever virus	0.46	4.24	298	19

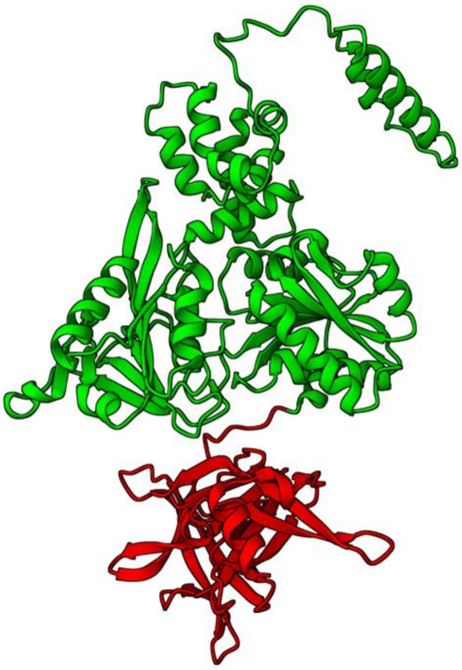

Figure S3. Tertiary structure model of HSTV NS3: NS3-helicase domain (green) and NS3-protease domain (red)

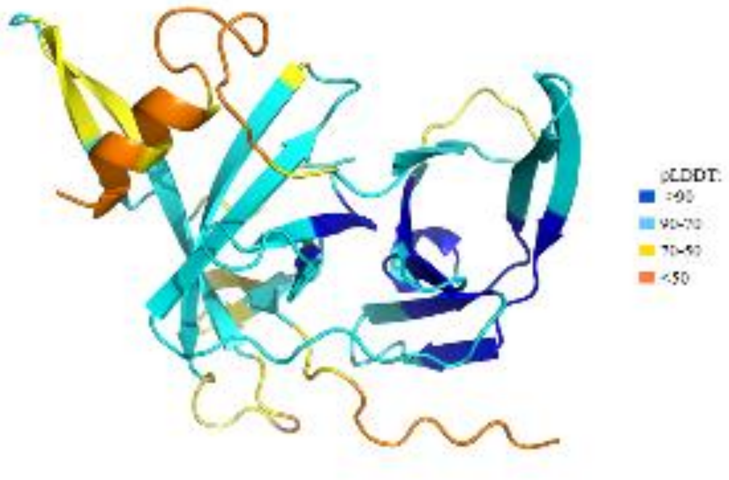

Figure S4. Tertiary structure model of HSTV NS3 protease in pLDDT color.

Table S4. Comparison of amino acid sequences and tertiary structure of HSTV NS3 protease domain with NS3pro domain of *Flaviviridae* family viruses.

**NS3pro PDB ID**	**Name of virus**	**TM-score**	**RMSD, Å**	**Aligned residues, a.a.**	**Amino acid sequence identity, %**
2FOM	Dengue virus 2	0.79	2.33	137	23
6MO0	Dengue virus 2	0.71	2.87	123	21
4M9F	Dengue virus 2	0.70	2.74	145	23
5H6V	Zika virus	0.75	2.82	137	21
5LC0	Zika virus	0.73	3.08	133	20
5TFO	Zika virus	0.70	3.46	127	18
6URV	Yellow fever virus	0.69	2.84	133	15
8CO8	West Nile virus	0.65	2.76	149	14
2F9U	Hepatitis C virus	0.69	4.64	118	17
5EPN	Hepatitis C virus	0.67	5.09	122	15
2A4Q	Hepatitis C virus	0.65	4.38	116	13
6DIT	Hepatitis C virus	0.60	4.36	127	11

5WX1	Classical swine fever virus	0.60	3.22	138	22

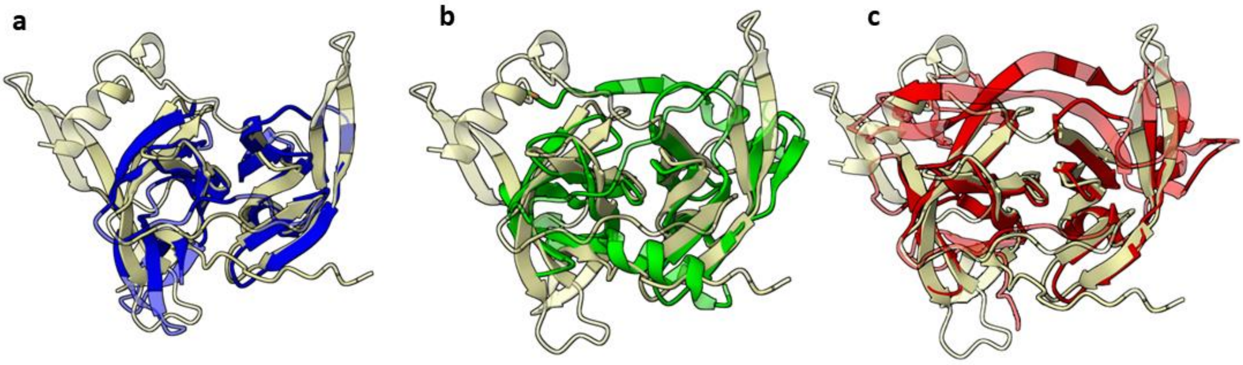

Figure S5. Imposition models of spatial structures HSTV NS3pro (ivory) with: (a) Dengue 2 virus, PDB ID: 2FOM (blue); (b) Hepatitis C virus, PDB ID: 2F9U (green); (c) Classical swine fever virus, PDB ID: 5WX1 (red).

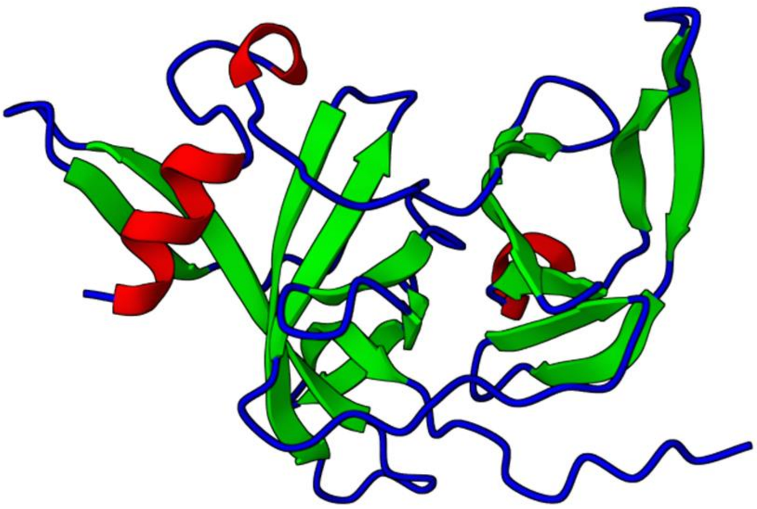

Figure S6. The model of spatial structure of HSTV NS3pro: α-helix (red), β-strand (green).

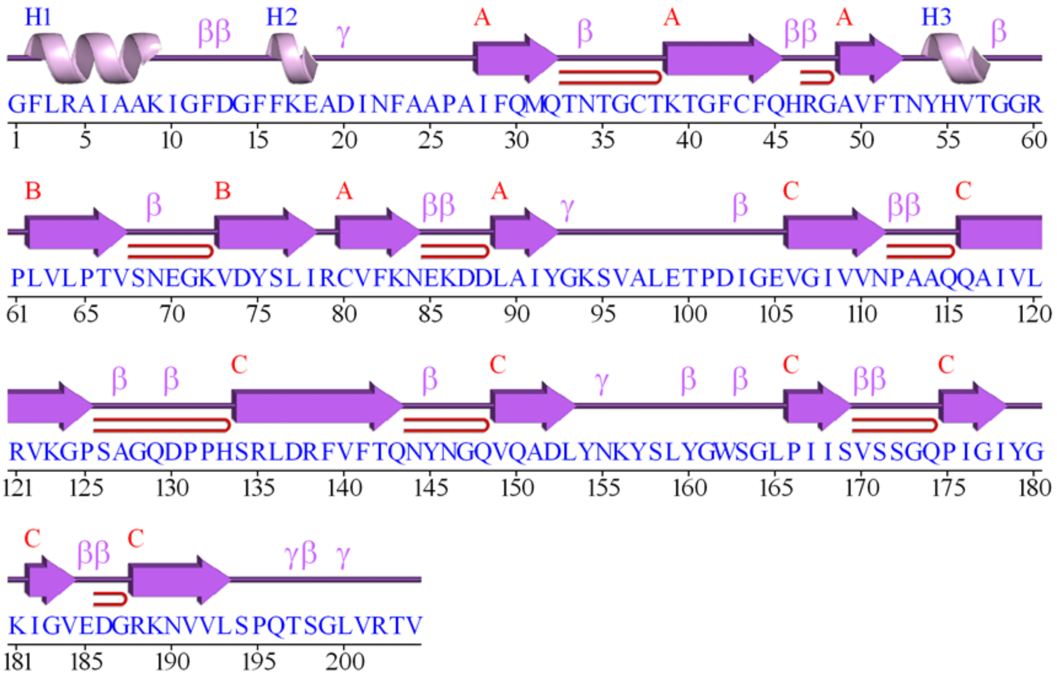

Figure S7. Secondary structure of HSTV NS3pro. Secondary structures: α-helices (H1, H2, etc) and strands (A, B, etc). Strand Motifs: β-beta turn; γ-gamma turn, and red beta hairpin

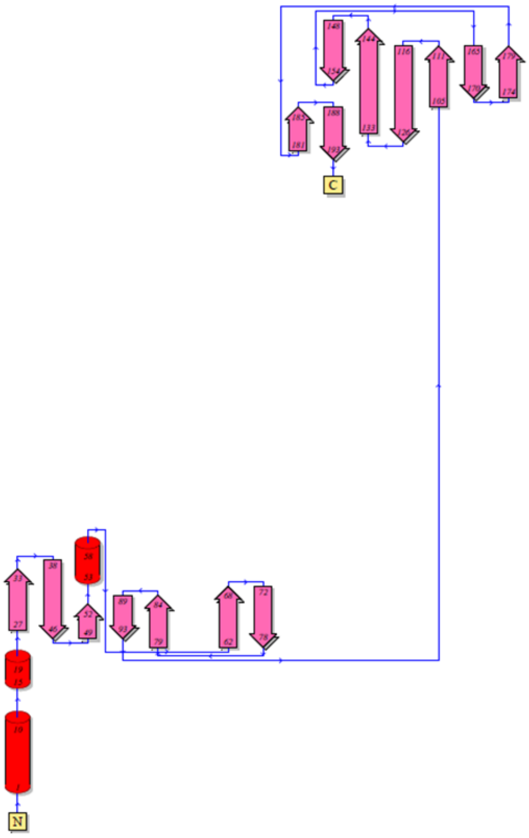

Figure S8. Topology diagram of HSTV NS3pro: α-helix (red), β-strands (pink), N-amino-terminus, C-carboxyl-terminus.

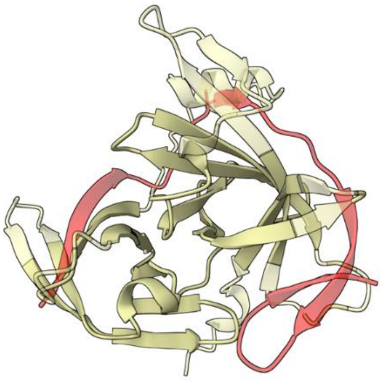

Figure S9. Model of spatial structure of HSTV NS3pro (ivory) in complex with NSTR1 extracellular domain (red).

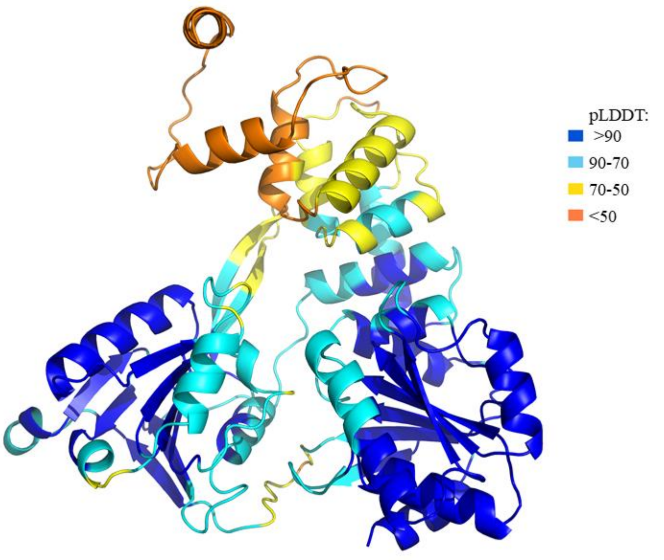

Figure S10. Tertiary structure model of HSTV NS3 helicase in pLDDT color.

Table S5. Comparison of amino acid sequences and tertiary structure of HSTV NS3hel domains with NS3hel domains of *Flaviviridae* family viruses.

NS3hel PDB ID	Name of virus	TM-score	RMSD, Å	Aligned residues, a.a.	Amino acid sequence identity, %
**D3 NS3hel HSTV**					
3KQL	Hepatitis C virus	0.31	6.18	40	6
2JLS	Dengue virus4	0.36	5.7	57	5
7V4Q	Kyasanur Forest disease virus	0.39	5.49	70	6
2V8O	Murray valley encephalitis virus	0.39	5.76	60	7
5K8L	Zika virus	0.42	5.18	68	8
7NXU	Tick-borne encephalitis virus	0.40	5.4	67	3
1YKS	Yellow fever virus	0.39	5.22	68	8
4CBL	Classical swine fever virus	0.35	5.79	59	8
5WSO	Bovine viral diarrhea virus	0.35	5.7	53	3
**D1-D2 NS3hel HSTV**					
3KQL	Hepatitis C virus	0.74	2.89	262	23
2JLS	Dengue virus4	0.74	3.26	258	17
7V4Q	Kyasanur Forest disease virus	0.72	3.46	247	20
2V8O	Murray valley encephalitis virus	0.71	3.63	242	16
5K8L	Zika virus	0.72	3.42	244	19
7NXU	Tick-borne encephalitis virus	0.70	3.34	250	21
1YKS	Yellow fever virus	0.68	3.56	243	19
4CBL	Classical swine fever virus	0.61	05.02	183	15
5WSO	Bovine viral diarrhea virus	0.57	4.82	175	16
**NS3hel HSTV**					
2JLS	Dengue virus4	0.63	4.38	291	14
2WZQ	Dengue virus4	0.63	4.44	285	14
8GZQ	Dengue virus3	0.6	4.38	291	14
5VI7	Zika virus	0.63	4.13	305	15

7V2Z	Zika virus	0.62	4.52	290	16
7NXU	Tick-borne encephalitis virus	0.62	4.63	285	18
2V8O	Murray valley encephalitis virus	0.62	4.55	285	15
7V4Q	Kyasanur Forest disease virus	0.61	4.9	274	17
1YKS	Yellow fever virus	0.59	4.79	276	17
4CBL	Classical swine fever virus	0.63	4.45	287	18
5WSO	Bovine viral diarrhea virus	0.6	4.56	271	18
1A1V	Hepatitis C virus	0.66	3.46	323	20
4WXR	Hepatitis C virus	0.64	3.96	315	19
2ZJO	Hepatitis C virus	0.62	4.32	304	15

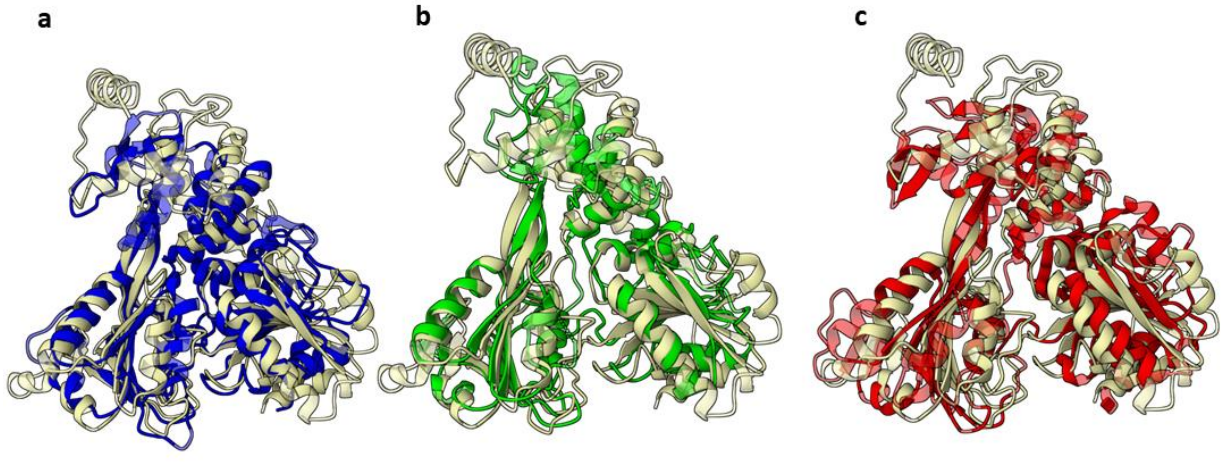

Figure S11. Imposition models of HSTV NS3 helicase tertiary structures (ivory) with: (a) Dengue 4 virus, PDB ID: 2JLS (blue); (b) Hepatitis C virus, PDB ID: 1A1V (green); (c) Classical swine fever virus, PDB ID: 4CBL (red).

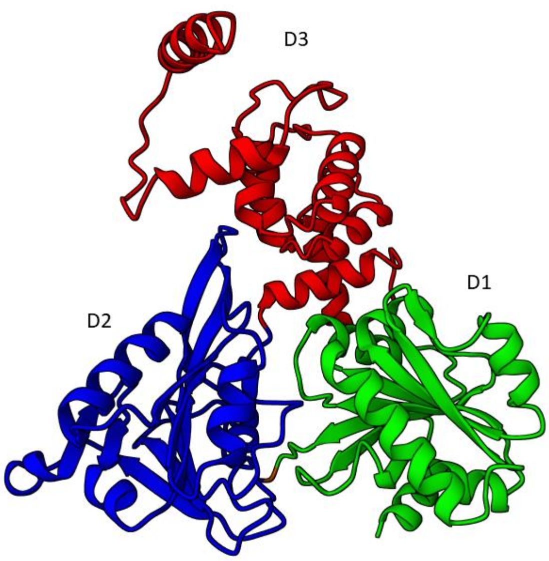

Figure S12. Domain organization of HSTV NS3 helicase spatial structure: domain 1 (green), domain 2 (blue), and domain 3 (red).

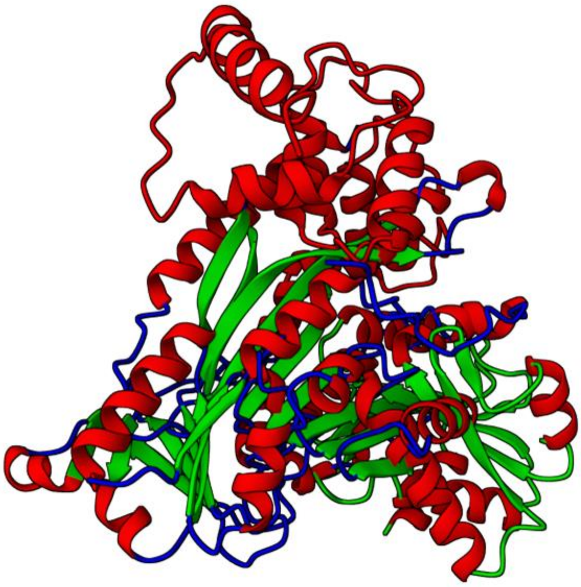

Figure S13. The model of spatial structure of HSTV NS3hel: α-helix (red), β-strand (green).

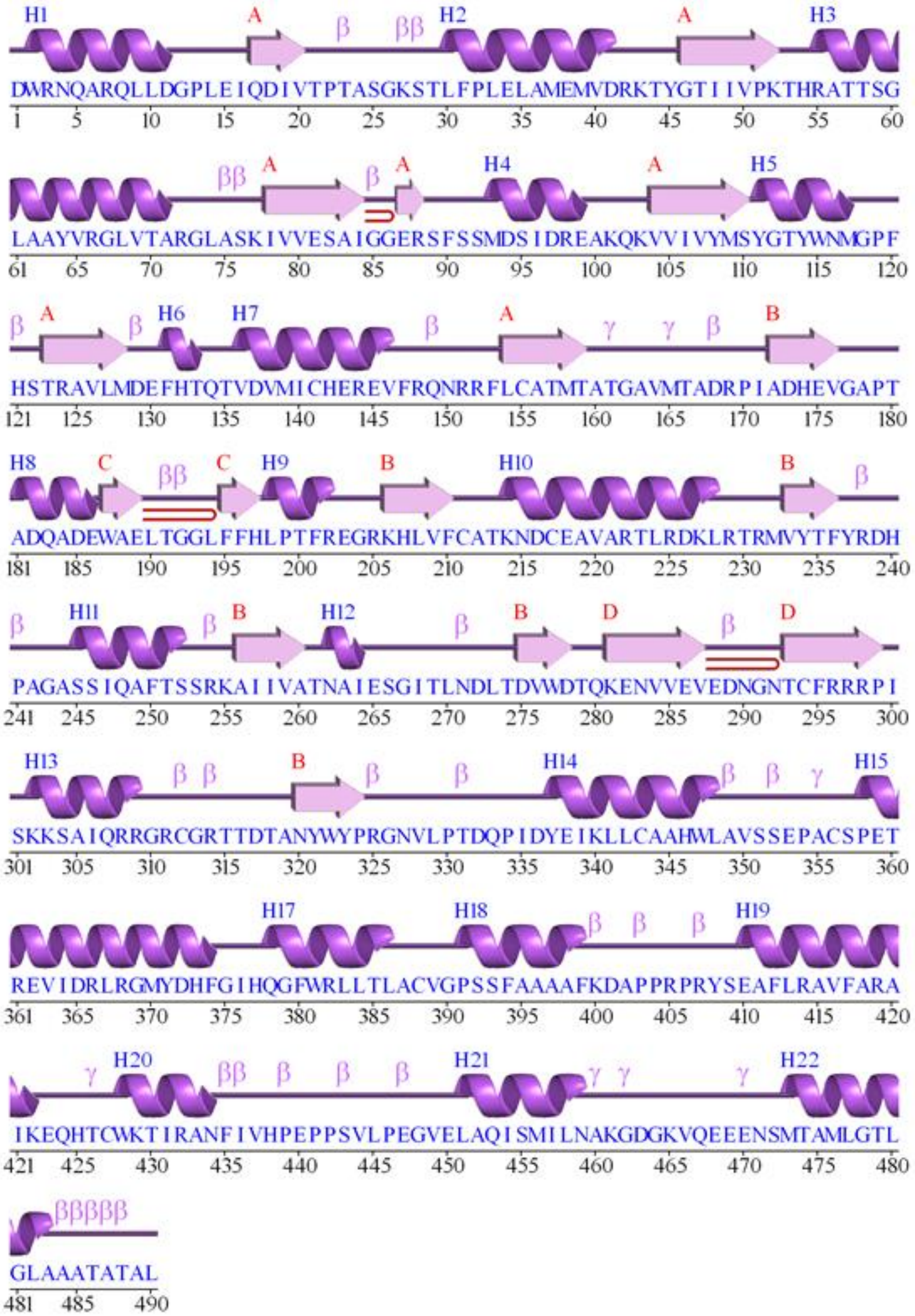

Figure S14. Secondary structure of HSTV NS3hel. Secondary structures: α-helices (H1, H2, etc) and strands (A, B, etc). Strand Motifs: β-beta turn; γ-gamma turn, and red beta hairpin.

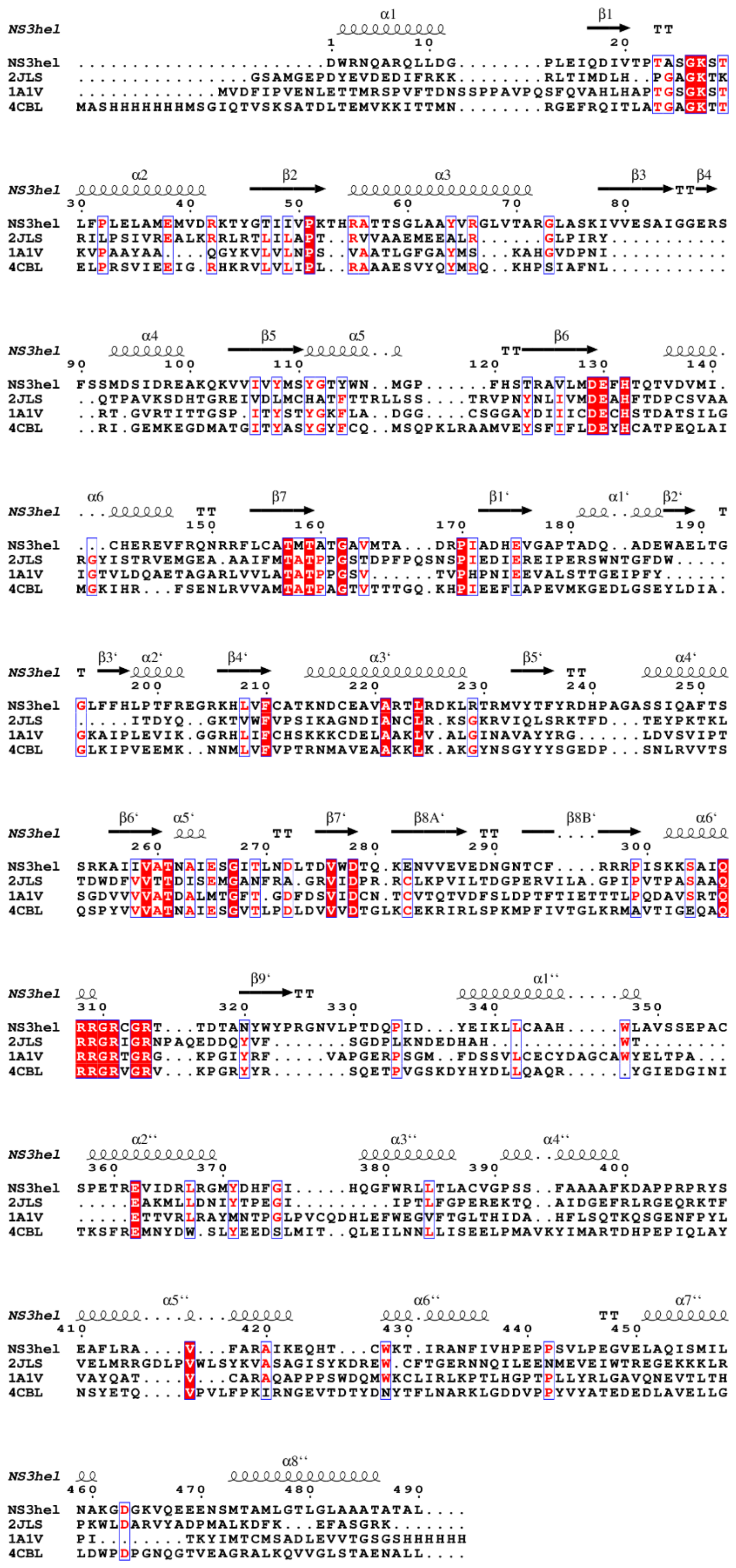

Figure S15. Sequence alignment of HSTV NS3hel with NS3hel Dengue 4 virus (2JLS), NS3hel Hepatitis C virus (1A1V), and NS3hel Classical swine fever virus (4CBL). Highly conserved amino acids are highlighted in red.

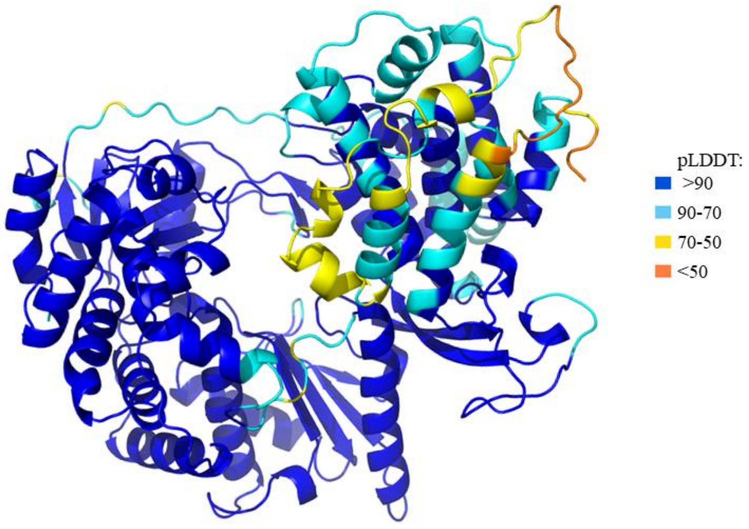

Figure S16. Tertiary structure model of HSTV NS5RdRp in pLDDT color.

Table S6. Comparison of amino acid sequences and tertiary structure of HSTV NS5 RdRp with NS5RdRp of *Flaviviridae* family viruses.

**NS5RdRp PDB ID**	**Name of virus**	**TM-score**	**RMSD, Å**	**Aligned residues, a.a.**	**Amino acid sequence identity, %**
7XD8	Dengue Virus 2	0.72	3.26	498	13%
4C11	Dengue Virus 3	0.68	3.58	476	12%
6IZZ	Dengue Virus 3	0.67	3.25	471	13%
5U0C	Zika virus	0.72	3.61	494	15%
6I7P	Zika virus	0.72	3.27	490	15%
5WZ3	Zika virus	0.66	3.58	456	14%
7ZIU	Ntaya virus	0.70	3.43	476	14%

7D6N	Tick-borne encephalitis virus	0.69	3.5	473	12%
4HDH	Japanese encephalitis virus	0.70	3.75	481	15%
7EKJ	Classical swine fever virus	0.66	3.54	476	18%
2CJQ	Bovine viral diarrhea virus	0.66	3.69	477	18%
6GP9	Hepatitis C virus	0.63	3.85	431	14%
1CSJ	Hepatitis C virus	0.63	3.91	426	12%
1YUY	Hepatitis C virus	0.63	4.17	420	14%

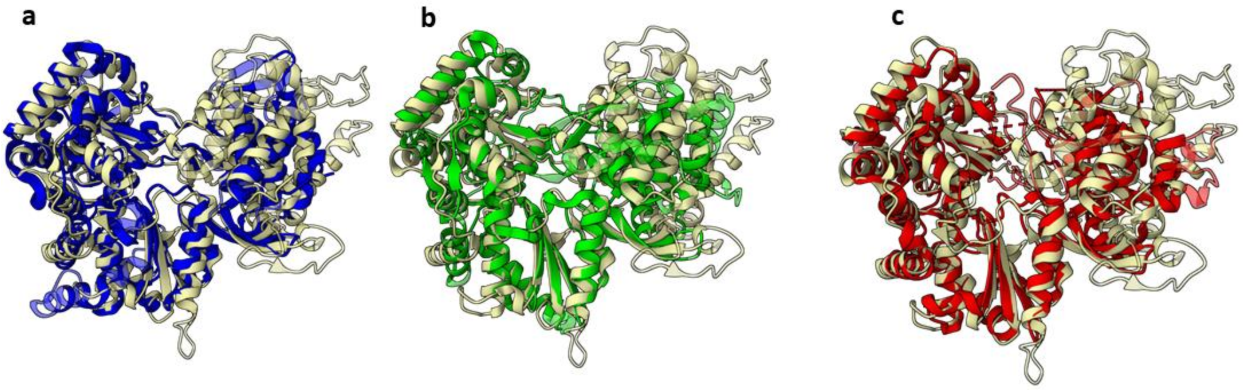

Figure S17. Imposition models of HSTV NS5RdRp spatial structures (ivory) with: (a) Dengue 2 virus, PDB ID: 7XD8 (blue); (b) Hepatitis C virus, PDB ID: 6GP9 (green); (c) Classical swine fever virus, PDB ID: 7EKJ (red).

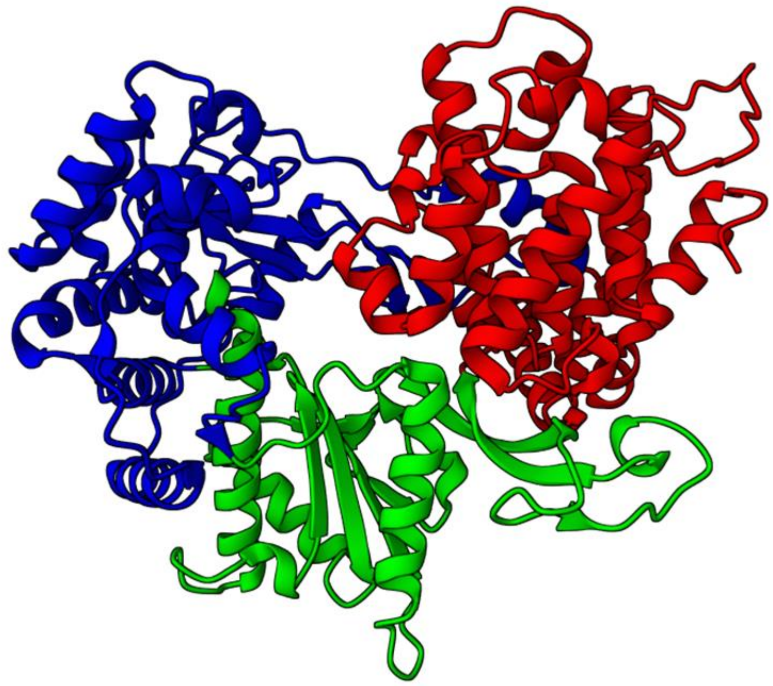

Figure S18. Domain organization of HSTV NS5RdRp spatial structure: thumb domain (red), palm domain (green), and fingers domain (blue).

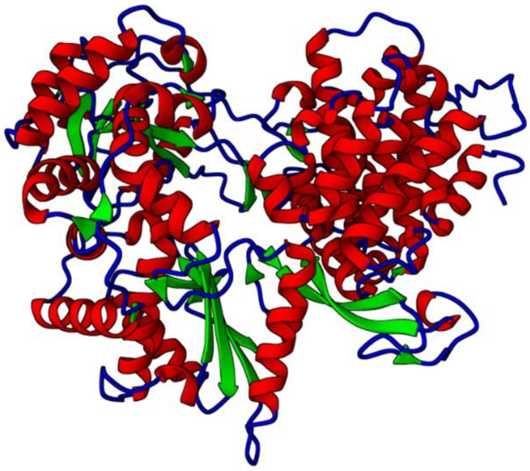

Figure S19. The model of spatial structure of HSTV NS5RdRp: α-helix (red), β-strand (green).

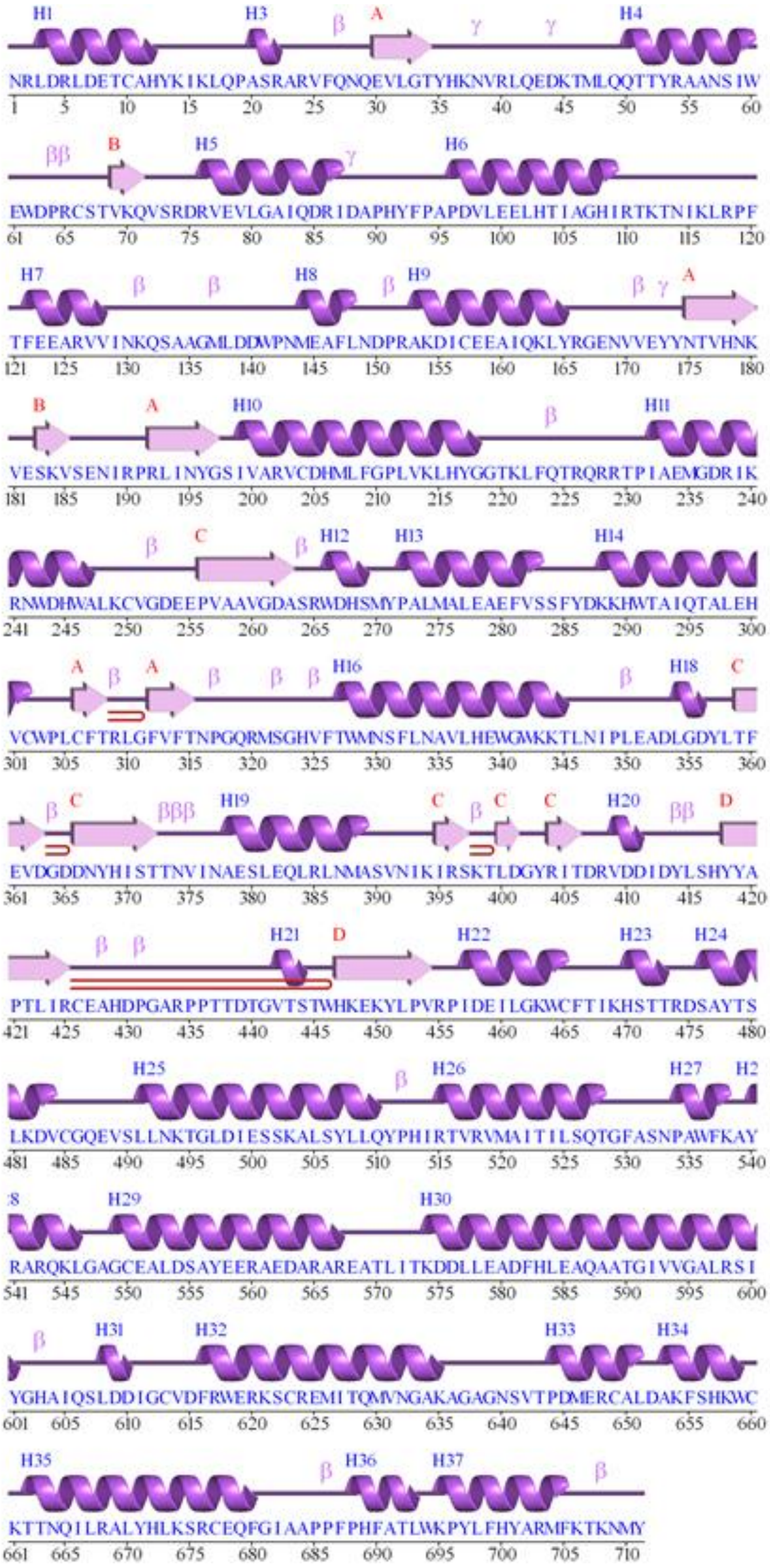

Figure S20. Secondary structure of HSTV NS5RdRp. Secondary structures: α-helices (H1, H2, etc) and strands (A, B, etc). Strand Motifs: β-beta turn; γ-gamma turn, and red beta hairpin.

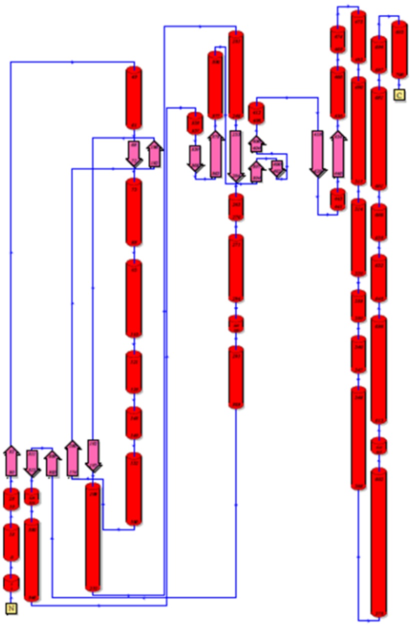

Figure S21. Topology diagram of HSTV NS5RdRp: α-helix (red), β-strands (pink), N-amino-terminus, C-carboxyl-terminus.

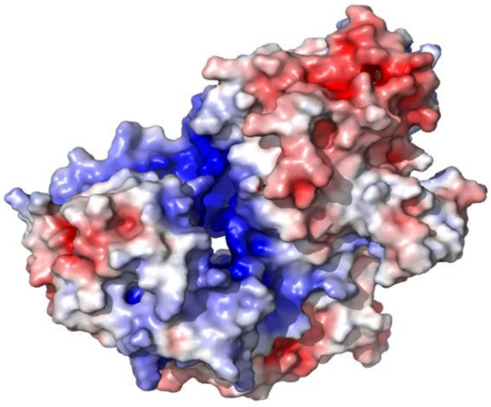

Figure S22. Electrostatic surface potential of HSTV NS5RdRp. The positive surface is colored blue, the negative surface is colored red.

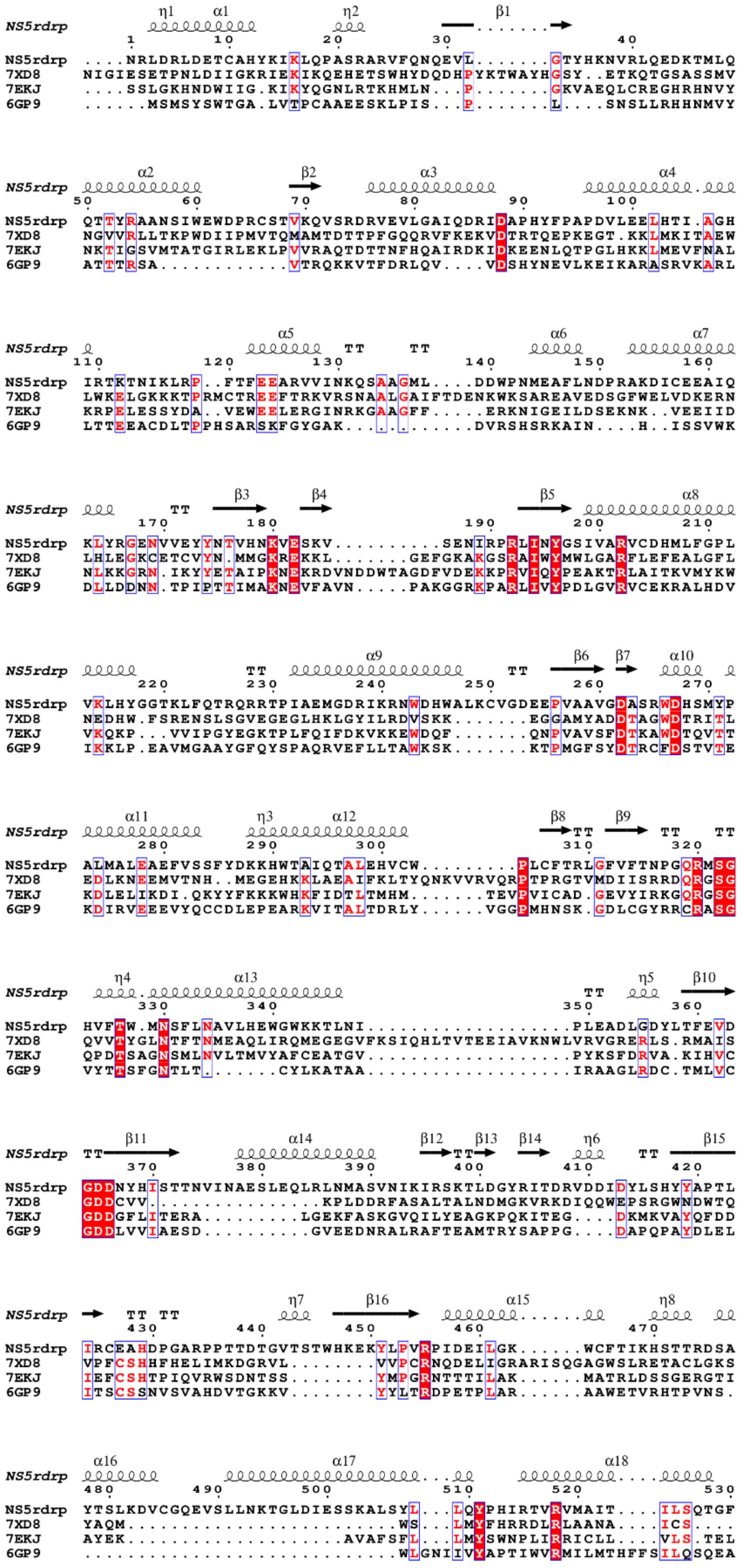

Figure S23. Sequence alignment of the HSTV NS5RdRp with NS5RdRp Dengue 2 virus (7XD8), NS5RdRp Classical swine fever virus (7EKJ), and NS5RdRp Hepatitis C virus (6GP9). Highly conserved amino acids are highlighted in red

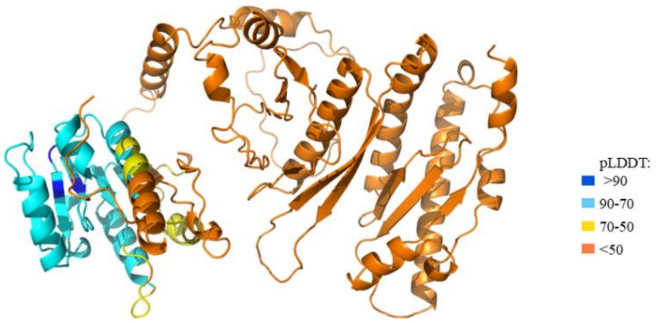

Figure S24. The model of spatial structure of HSTV NS5MTase-NS5X colored in pLDDT.

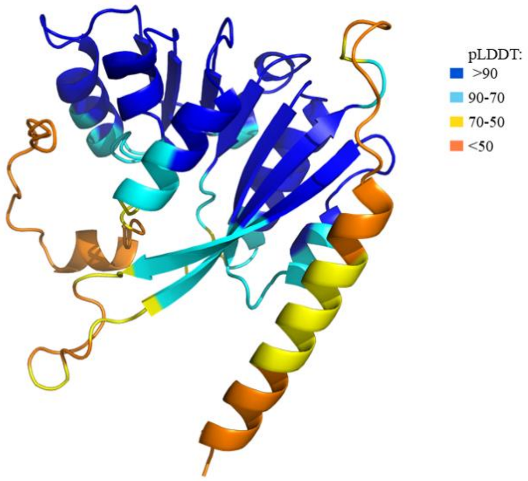

Figure S25. Tertiary structure model of HSTV NS5Mtase in pLDDT color.

Table S7. Comparison of amino acid sequences and tertiary structure of HSTV NS5MTase with NS5MTase of *Flaviviridae* family viruses.

MTase PDB ID	Name of virus	TM-score	RMSD, Å	Aligned residues, a.a.	Amino acid sequence identity, %
1WY7	Pyrococcus horikoshii	0.77	3.02	164	22
5WXB	Zika virus	0.49	3.99	141	13
5MRK	Zika virus	0.60	4.15	141	11
3P97	Dengue 3 virus	0.60	4.18	142	14
2OY0	West Nile virus	0.60	4.1	145	11
6QSN	Yellow fever virus	0.60	4.22	142	13
7D6M	Tick-borne encephalitis virus	0.60	4.21	143	11
8BXK	Ntaya virus	0.60	4.12	145	11
2WA1	Modoc Virus	0.59	3.69	142	12
8GY4	Alongshan virus	0.57	3.89	140	10

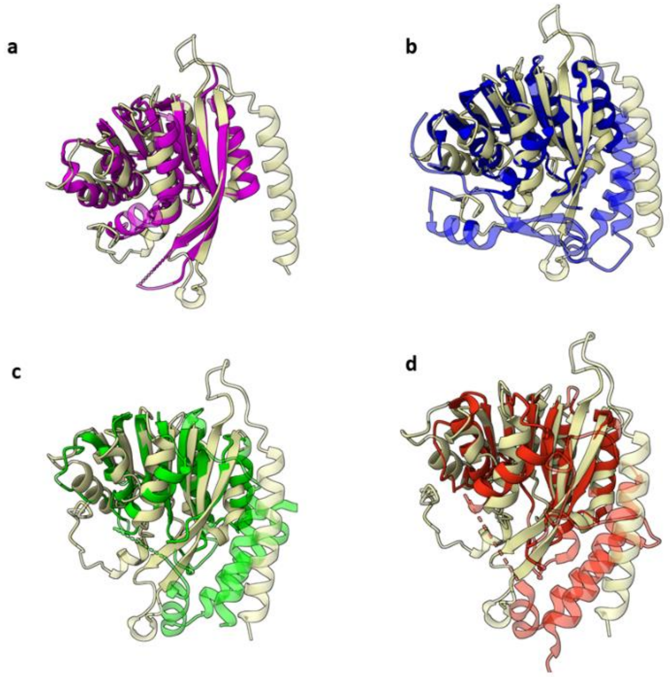

Figure S26. Imposition models of HSTV NS5Mtase spatial structures (ivory) with: (a) *Pyrococcus horikoshii*, PDB ID: 1WY7 (magenta); (b) Dengue 3 virus, PDB ID: 3P97 (blue); (c) Modoc Virus, PDB ID: 2WA1) (green); (d) Alongshan virus, PDB ID: 8GY4 (red).

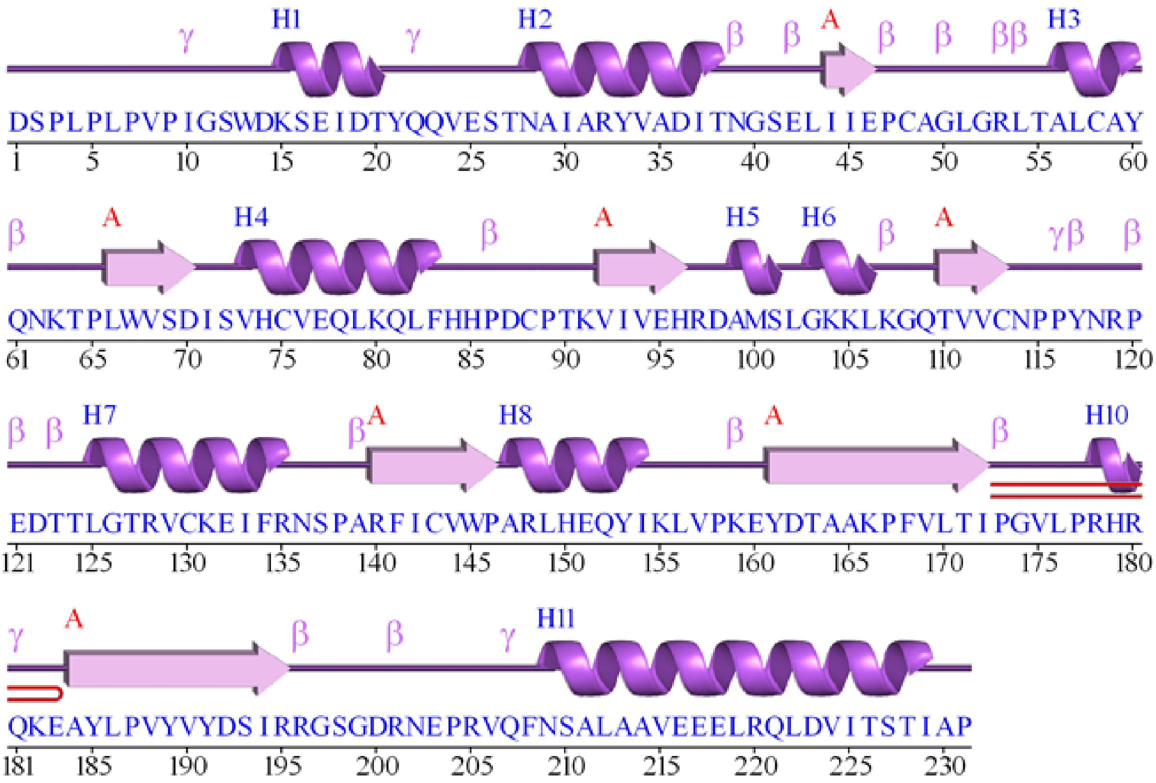

Figure S27. Secondary structure of HSTV NS5Mtase. Secondary structures: α-helices (H1, H2, etc) and strands (A, B, etc). Strand Motifs: β-beta turn; γ-gamma turn, and red beta hairpin.

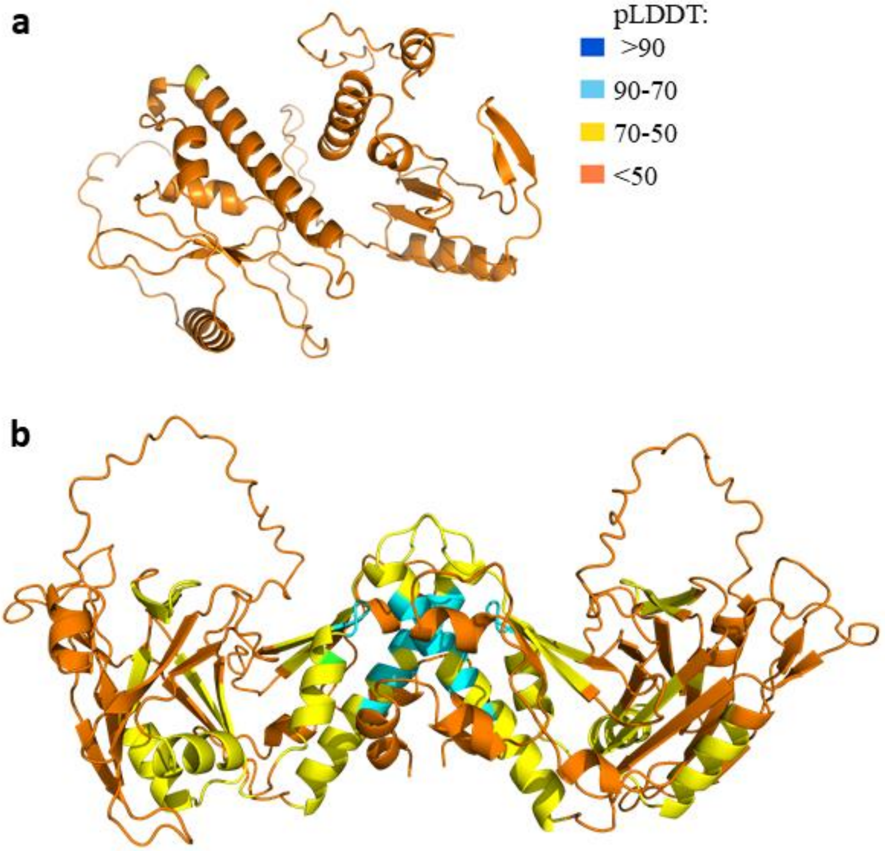

Figure S28. Tertiary structure model of HSTV NS5-X in pLDDT color: (a) NS5-X monomer, (b) NS5-X dimer.

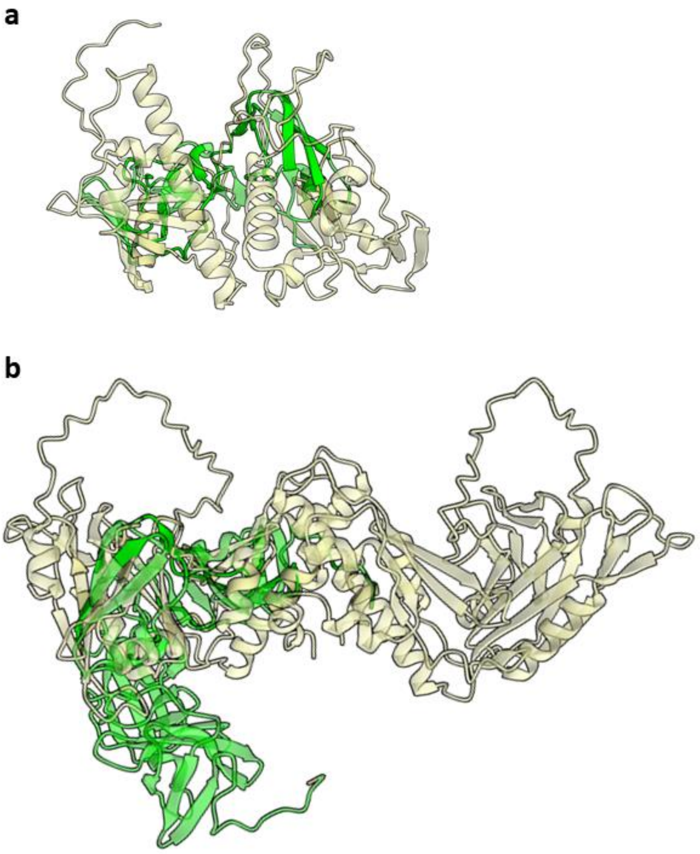

Figure S29. Imposition models of HSTV NS5-X spatial structures (ivory) with NS5A zinc-binding domain of Hepatitis C virus, PDB ID: 1ZH1 (green): (a) NS5-X monomer, (b) NS5-X dimer.

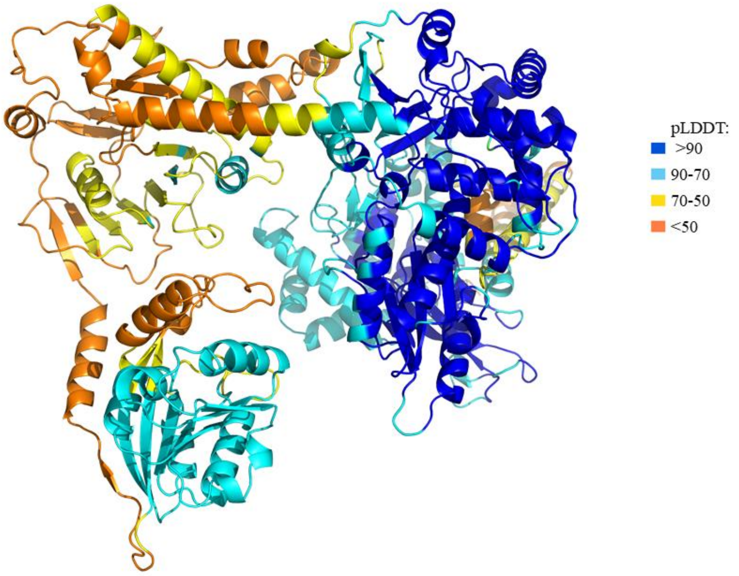

Figure S30. Tertiary structure model of HSTV NS5MTase-NS5X-NS5RdRp in pLDDT color.

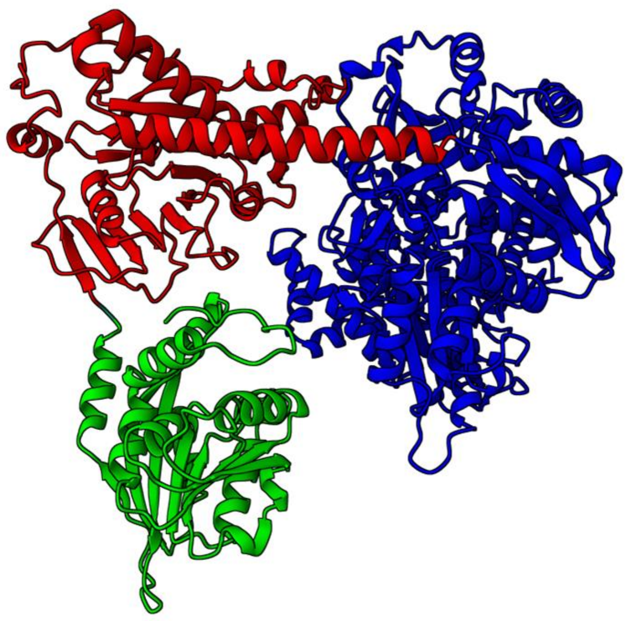

Figure S31. Domain organization of HSTV NS5MTase-NS5X-NS5RdRp spatial structure: NS5MTase (green), NS5-X (red), NS5RdRp (blue).
