# Supplementary figures and images for: Tertiary Structures of Haseki Tick Virus Nonstructural Proteins Are Similar to Those of Orthoflaviviruses
Source: Int J Mol Sci. 2024 Dec 20;25(24):13654. doi: 10.3390/ijms252413654 (PMC11678601; doi:10.3390/ijms252413654)

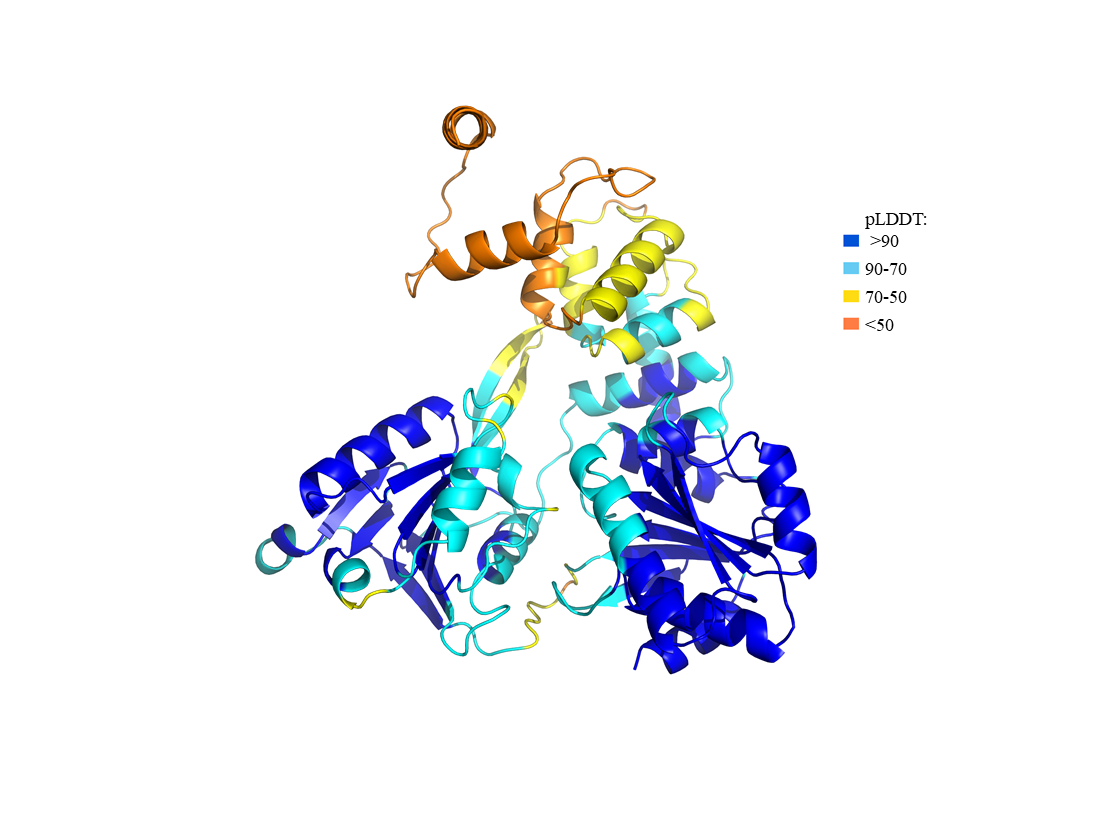

Supplement: Supplementary file 1 [file ijms-25-13654-s001.zip › Figure S10.TIF]

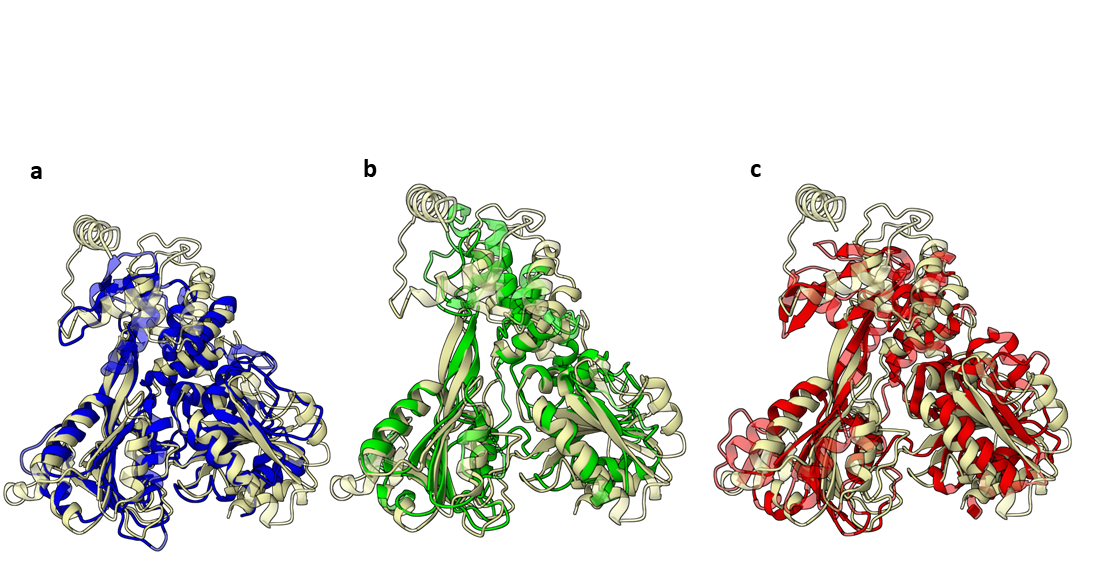

Supplement: Supplementary file 1 [file ijms-25-13654-s001.zip › Figure S11.TIF]

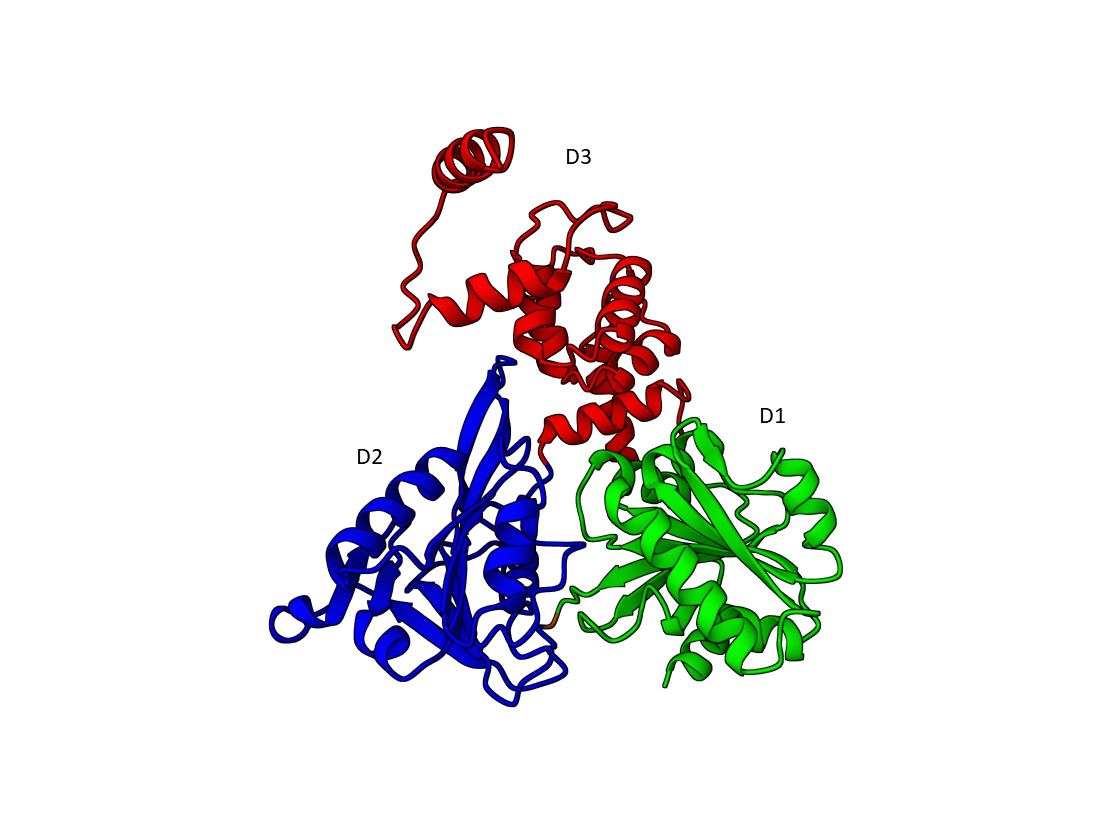

Supplement: Supplementary file 1 [file ijms-25-13654-s001.zip › Figure S12.TIF]

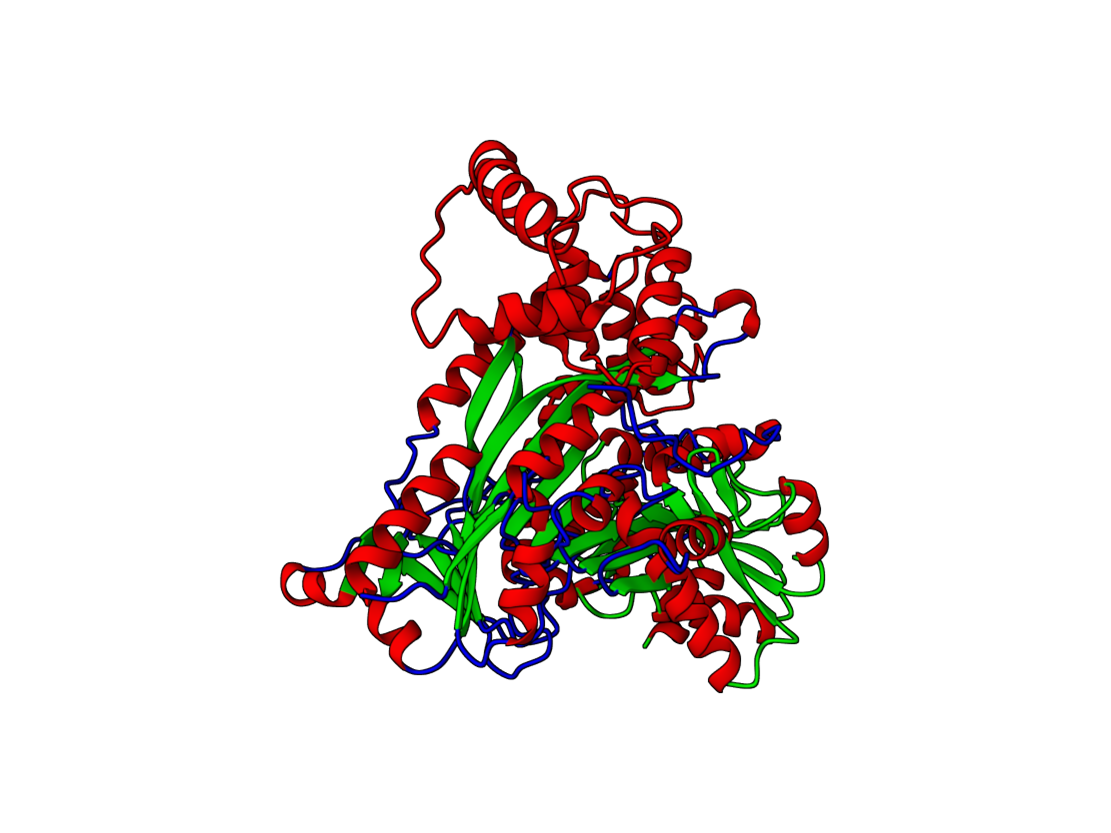

Supplement: Supplementary file 1 [file ijms-25-13654-s001.zip › Figure S13.TIF]

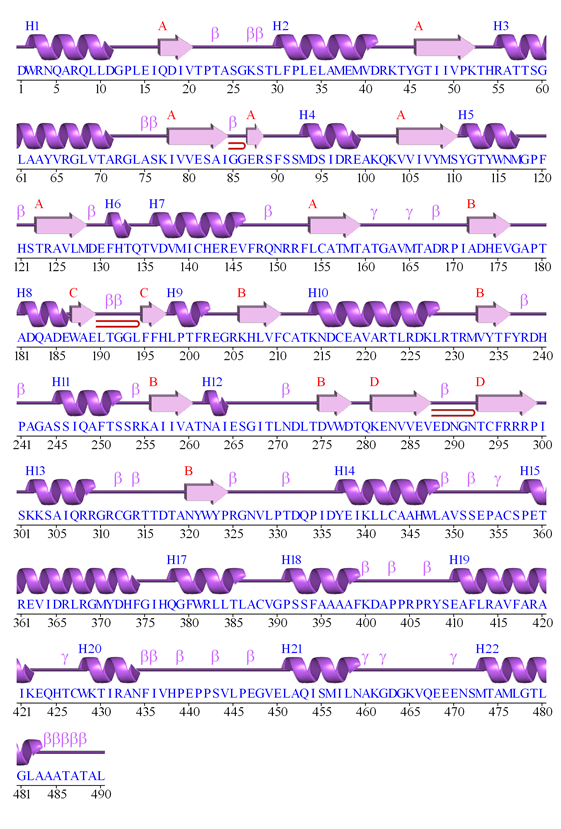

Supplement: Supplementary file 1 [file ijms-25-13654-s001.zip › Figure S14.TIF]

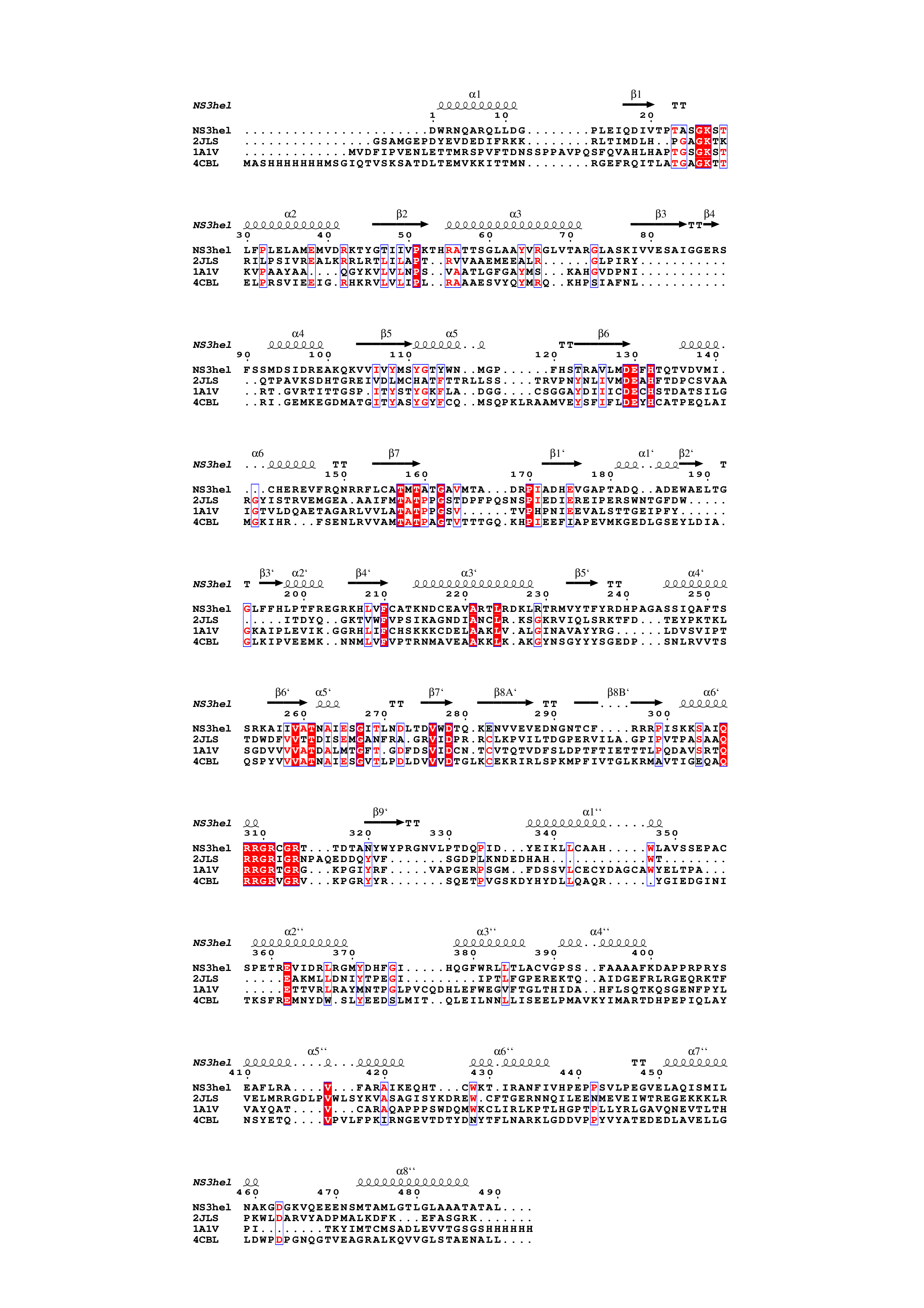

Supplement: Supplementary file 1 [file ijms-25-13654-s001.zip › Figure S15.tiff]

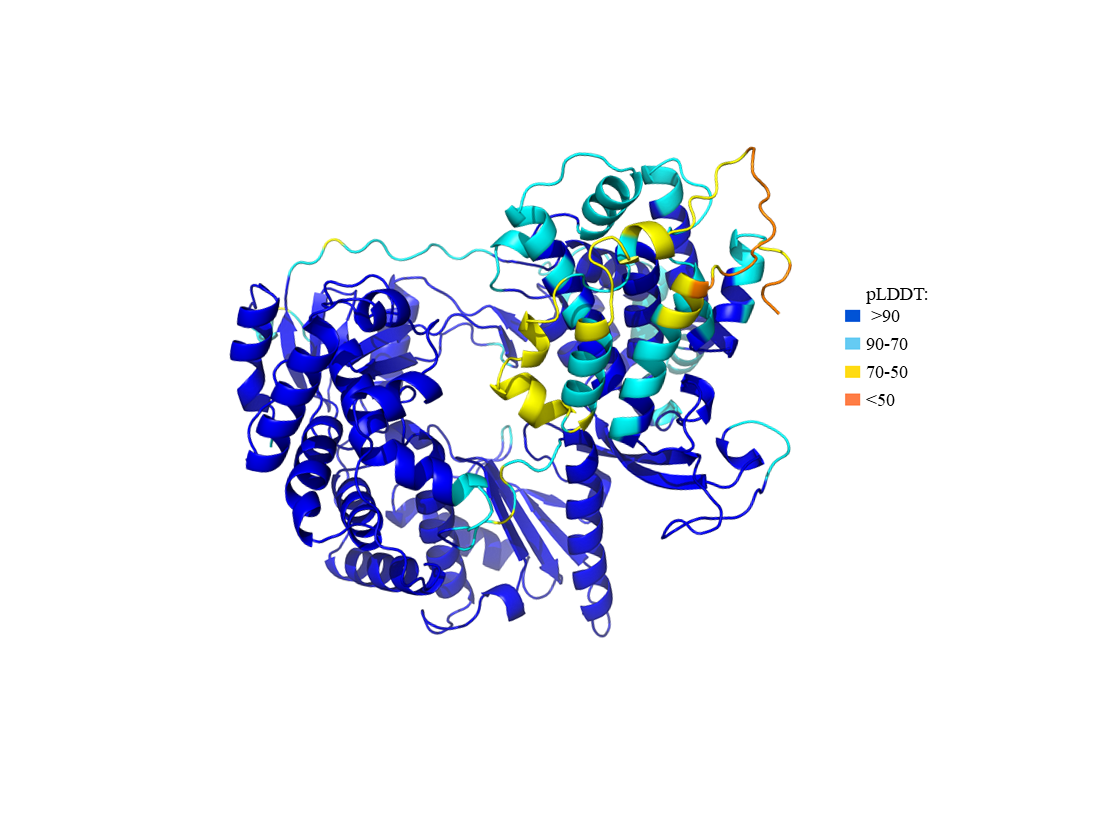

Supplement: Supplementary file 1 [file ijms-25-13654-s001.zip › Figure S16.TIF]

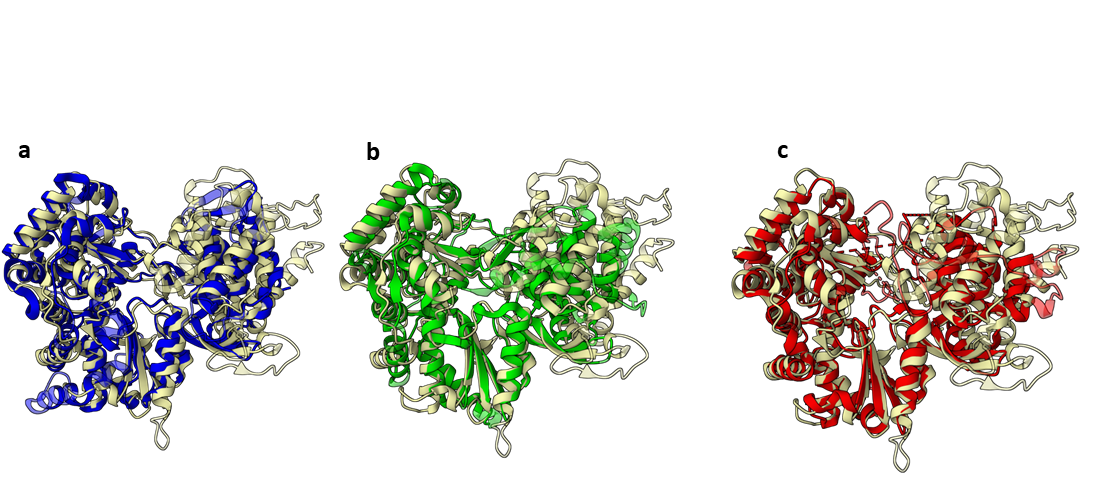

Supplement: Supplementary file 1 [file ijms-25-13654-s001.zip › Figure S17.TIF]

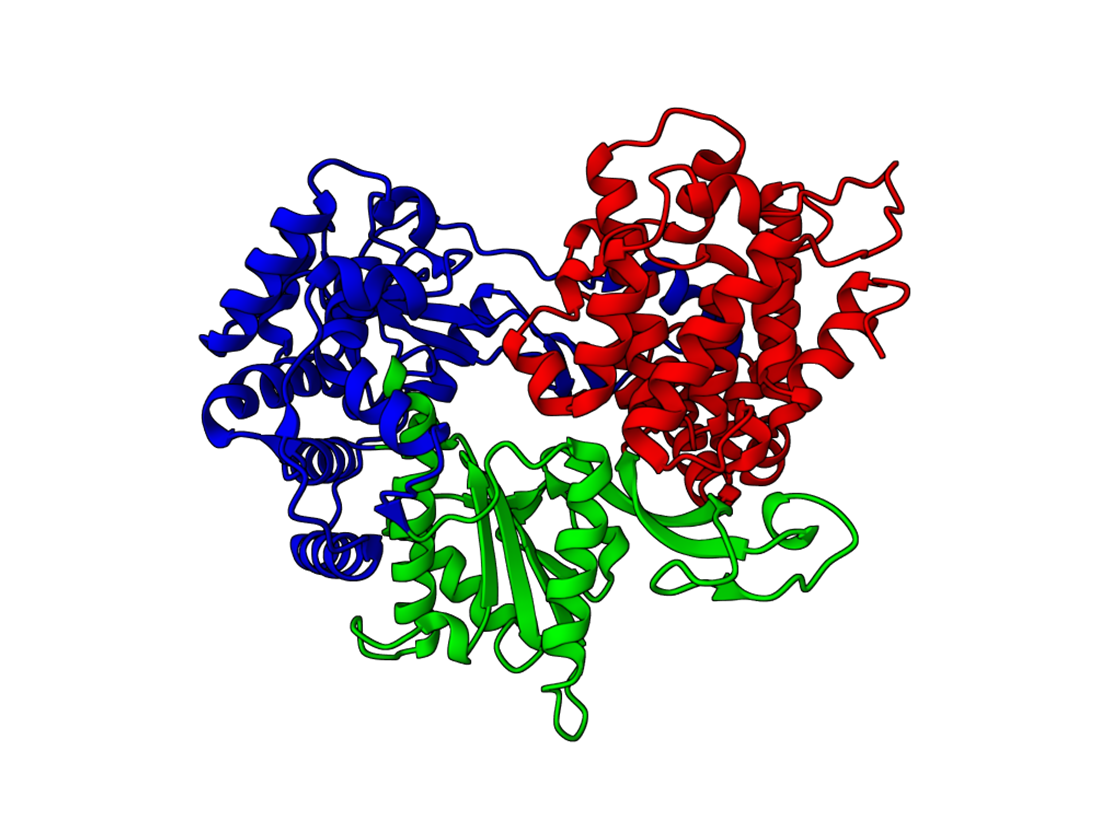

Supplement: Supplementary file 1 [file ijms-25-13654-s001.zip › Figure S18.TIF]

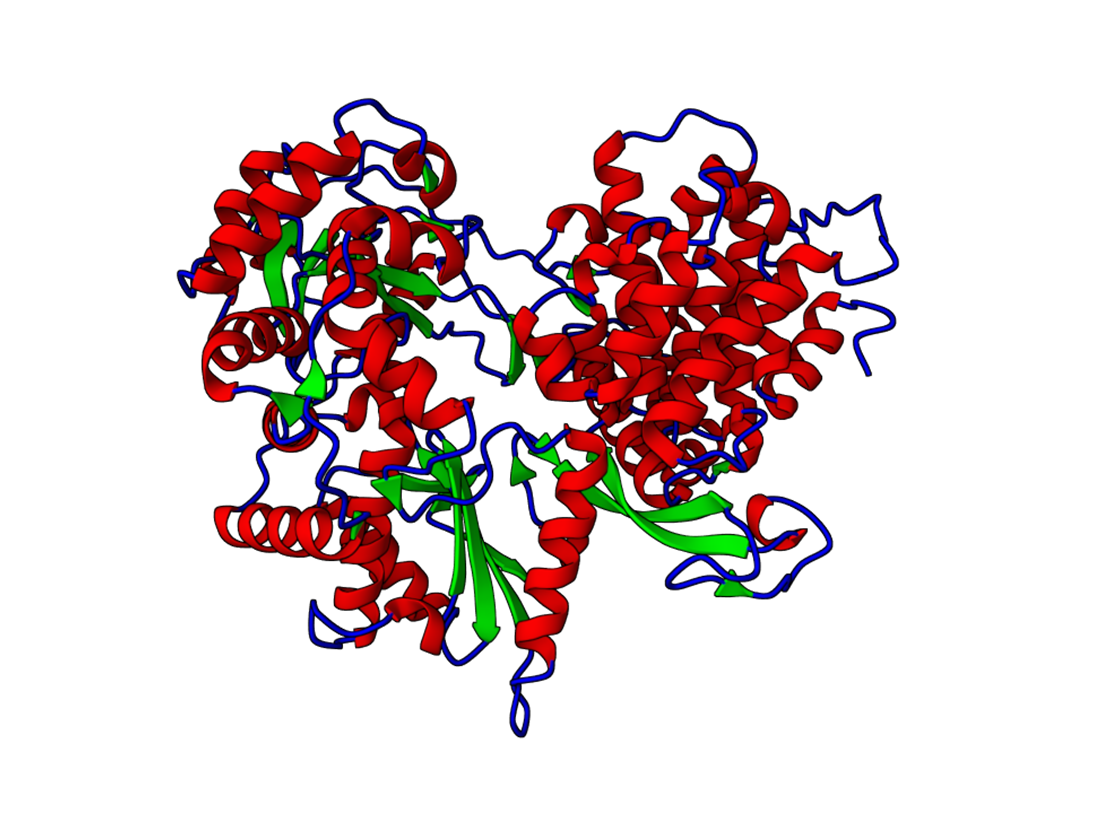

Supplement: Supplementary file 1 [file ijms-25-13654-s001.zip › Figure S19.TIF]

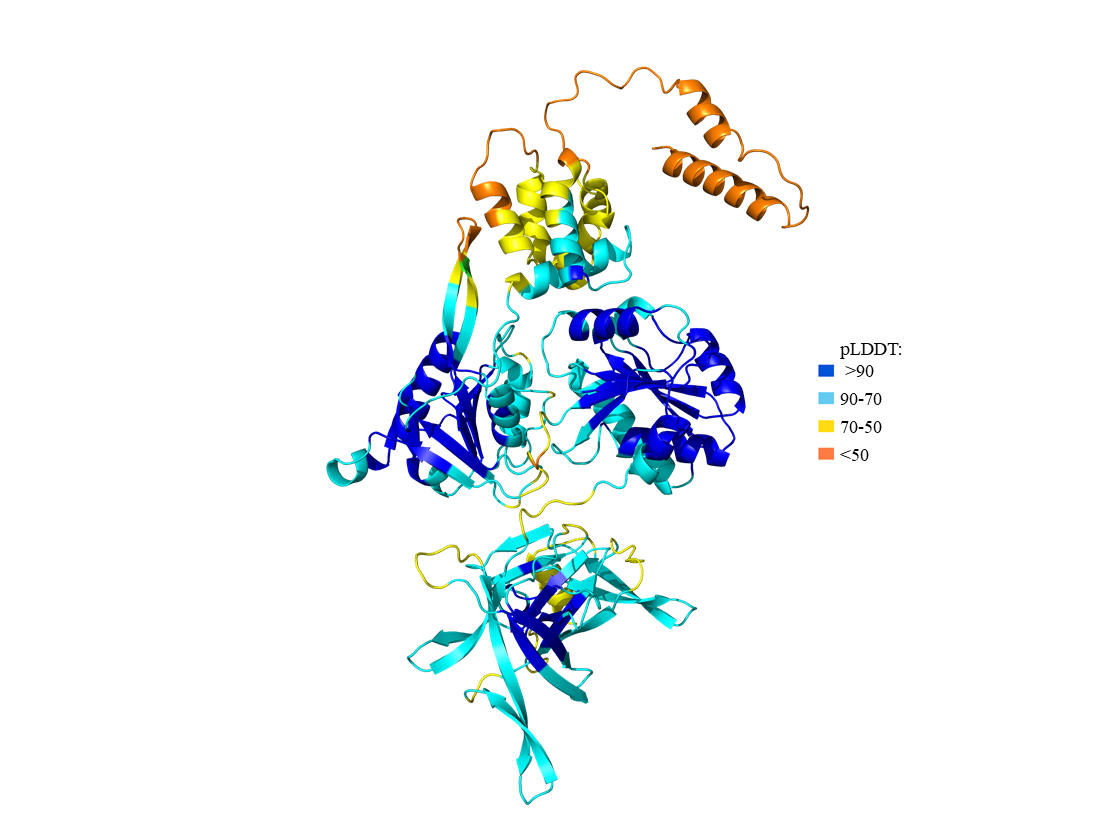

Supplement: Supplementary file 1 [file ijms-25-13654-s001.zip › Figure S2.TIF]

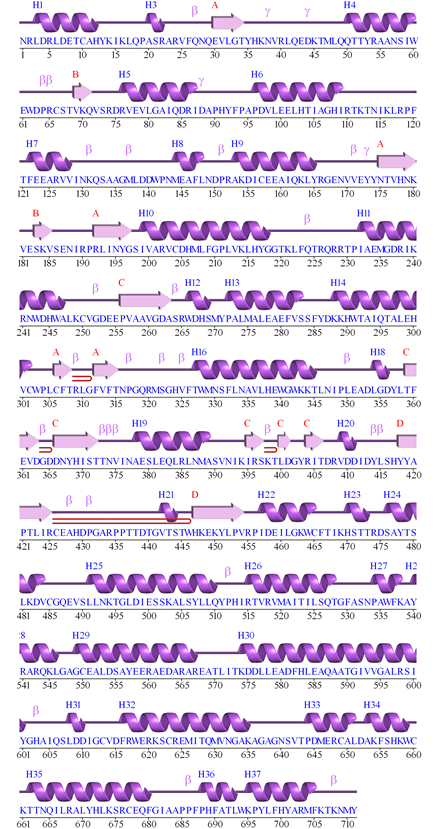

Supplement: Supplementary file 1 [file ijms-25-13654-s001.zip › Figure S20.TIF]

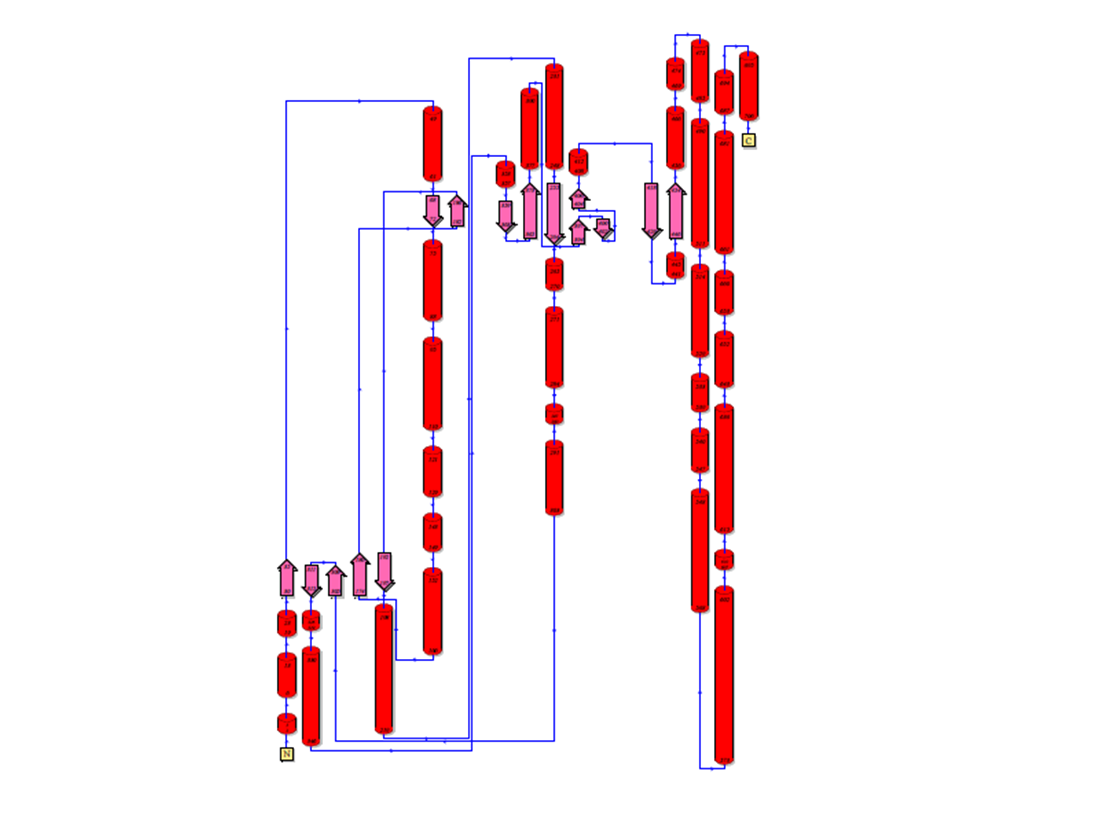

Supplement: Supplementary file 1 [file ijms-25-13654-s001.zip › Figure S21.TIF]

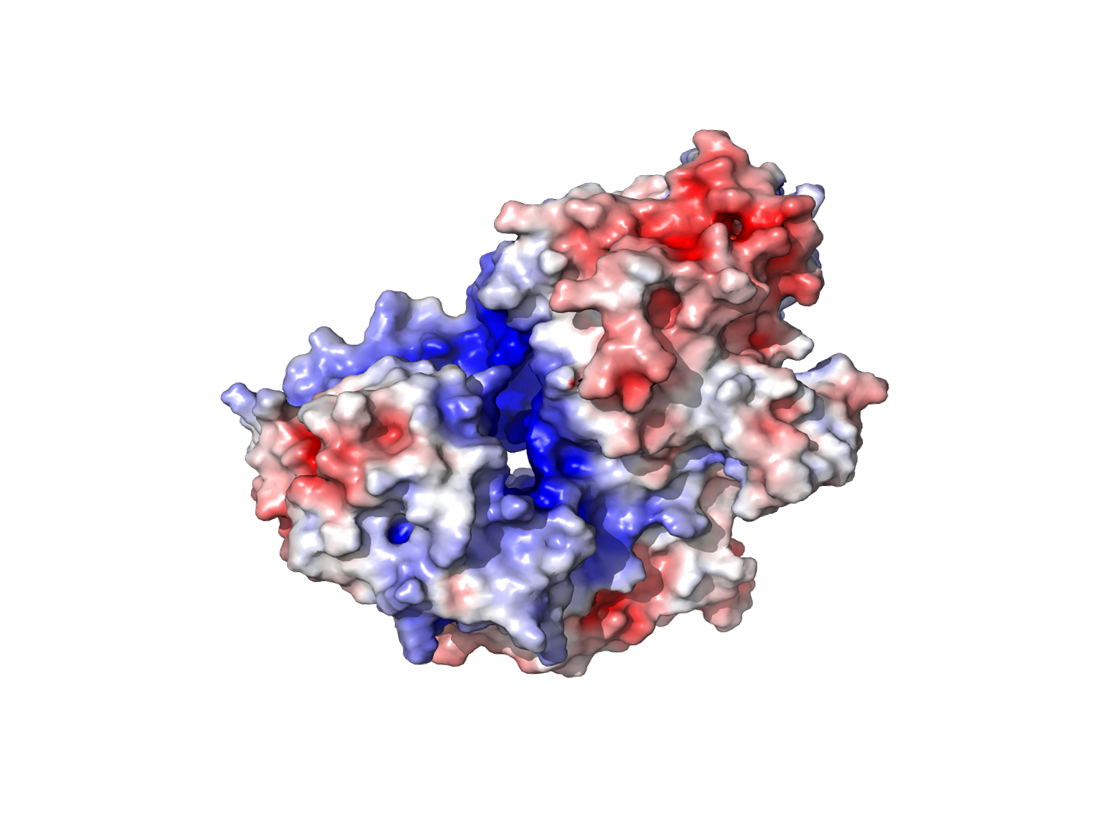

Supplement: Supplementary file 1 [file ijms-25-13654-s001.zip › Figure S22.TIF]

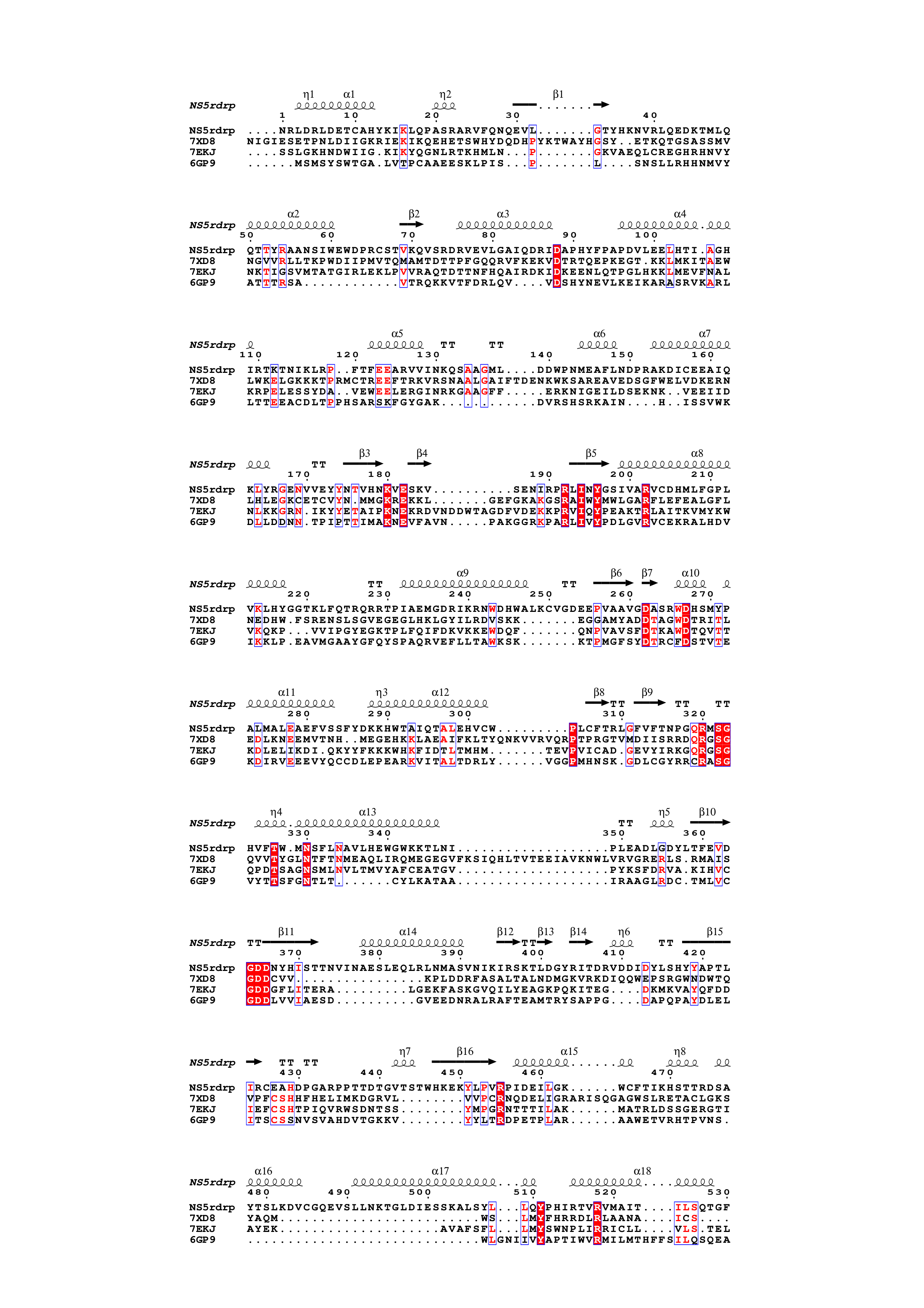

Supplement: Supplementary file 1 [file ijms-25-13654-s001.zip › Figure S23.tiff]

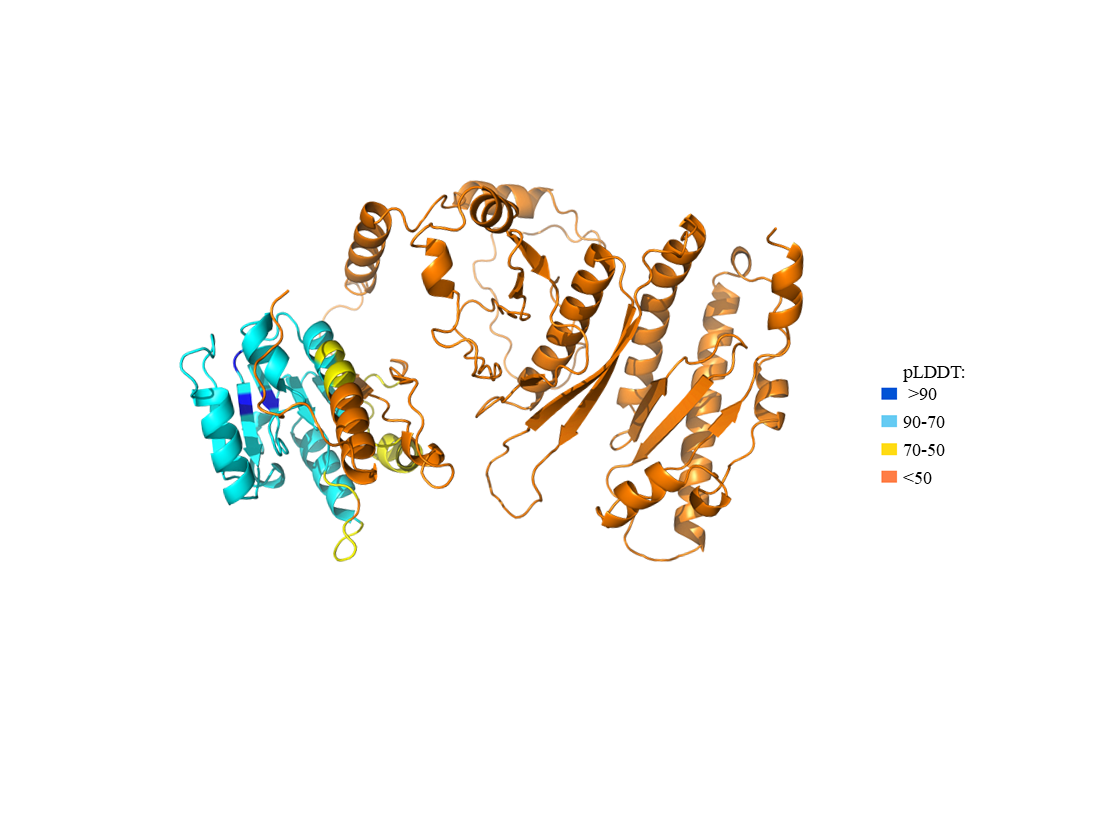

Supplement: Supplementary file 1 [file ijms-25-13654-s001.zip › Figure S24.TIF]

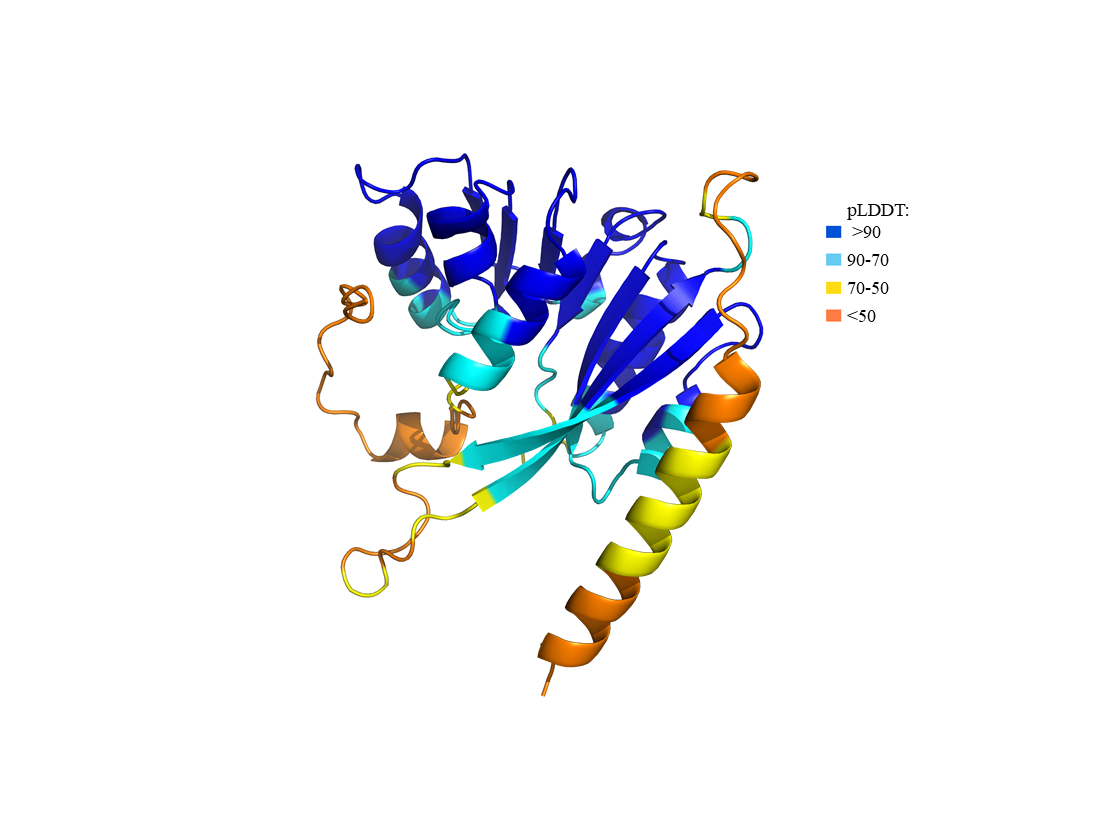

Supplement: Supplementary file 1 [file ijms-25-13654-s001.zip › Figure S25.TIF]

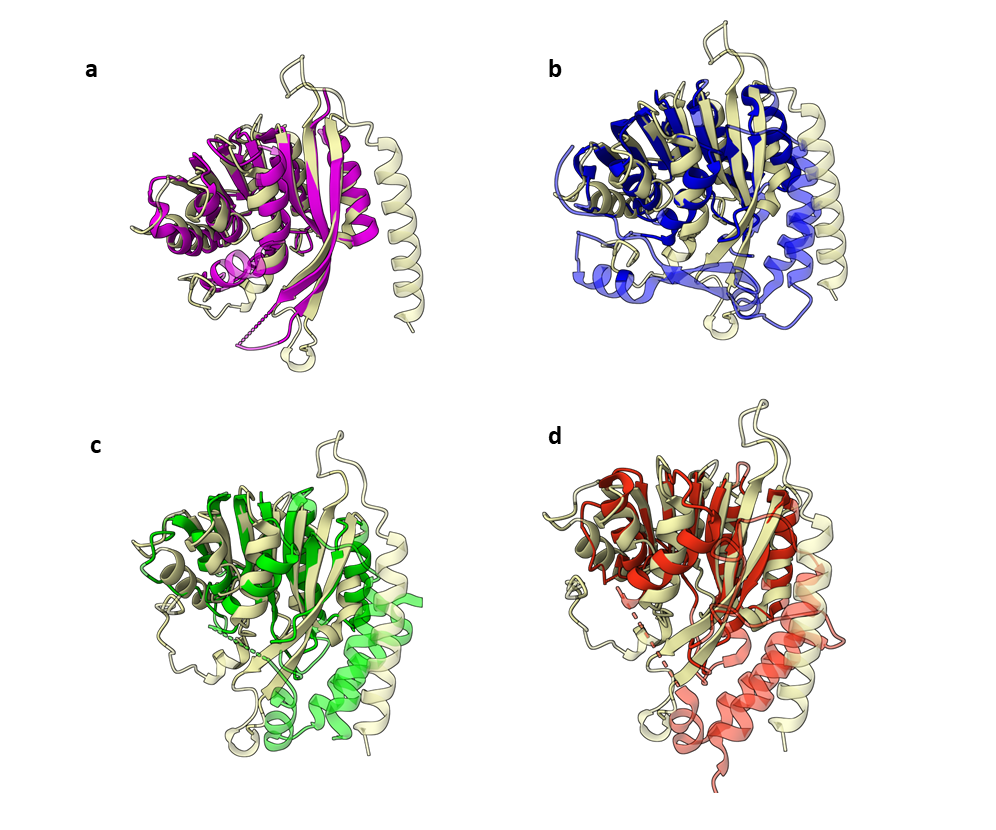

Supplement: Supplementary file 1 [file ijms-25-13654-s001.zip › Figure S26.TIF]

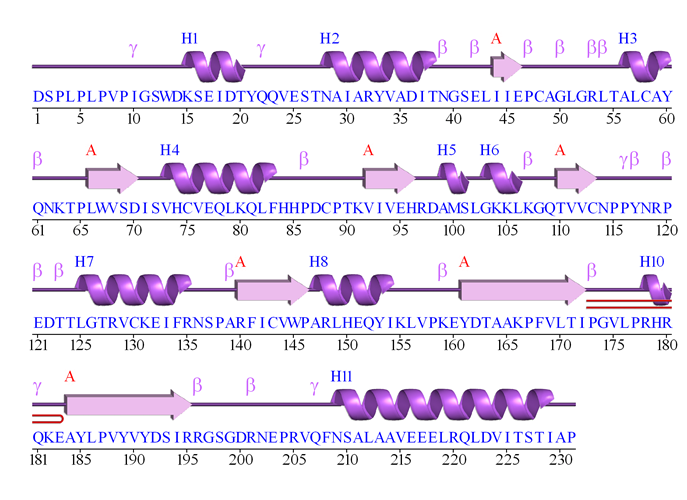

Supplement: Supplementary file 1 [file ijms-25-13654-s001.zip › Figure S27.TIF]

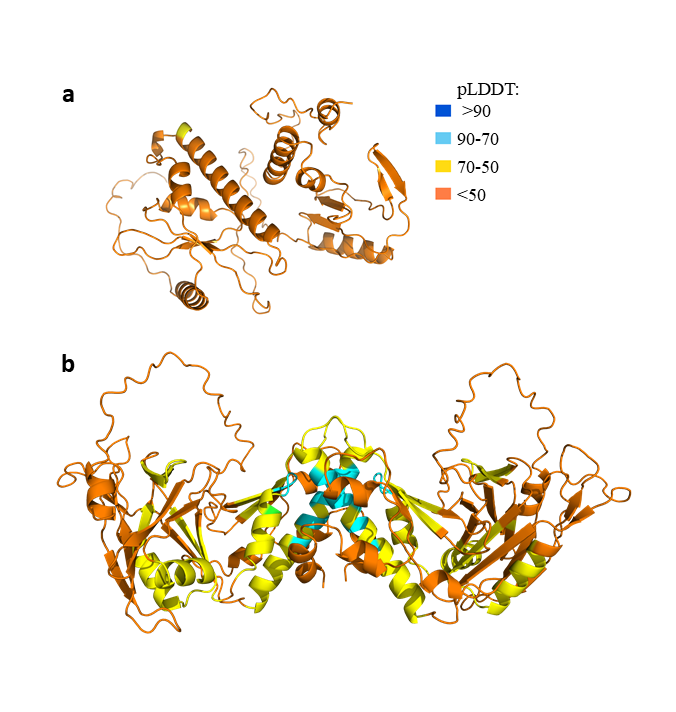

Supplement: Supplementary file 1 [file ijms-25-13654-s001.zip › Figure S28.TIF]

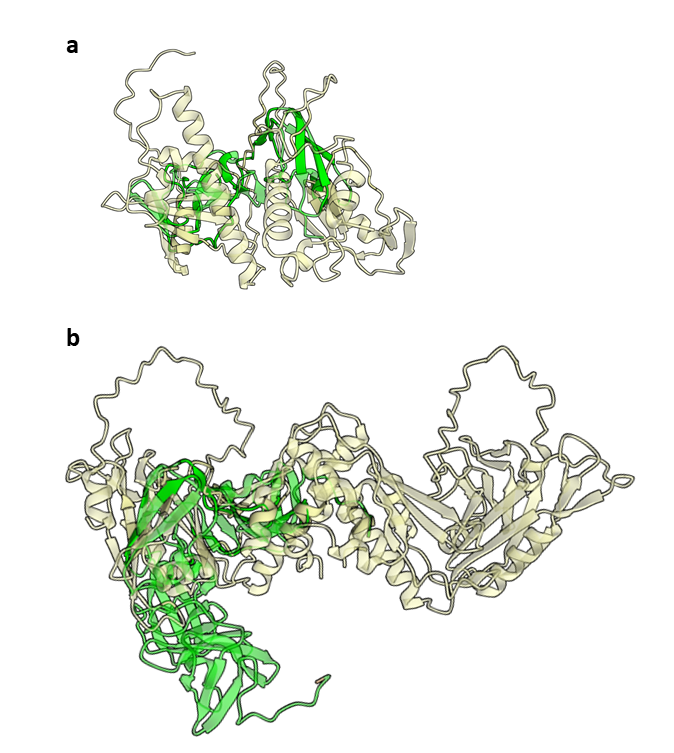

Supplement: Supplementary file 1 [file ijms-25-13654-s001.zip › Figure S29.TIF]

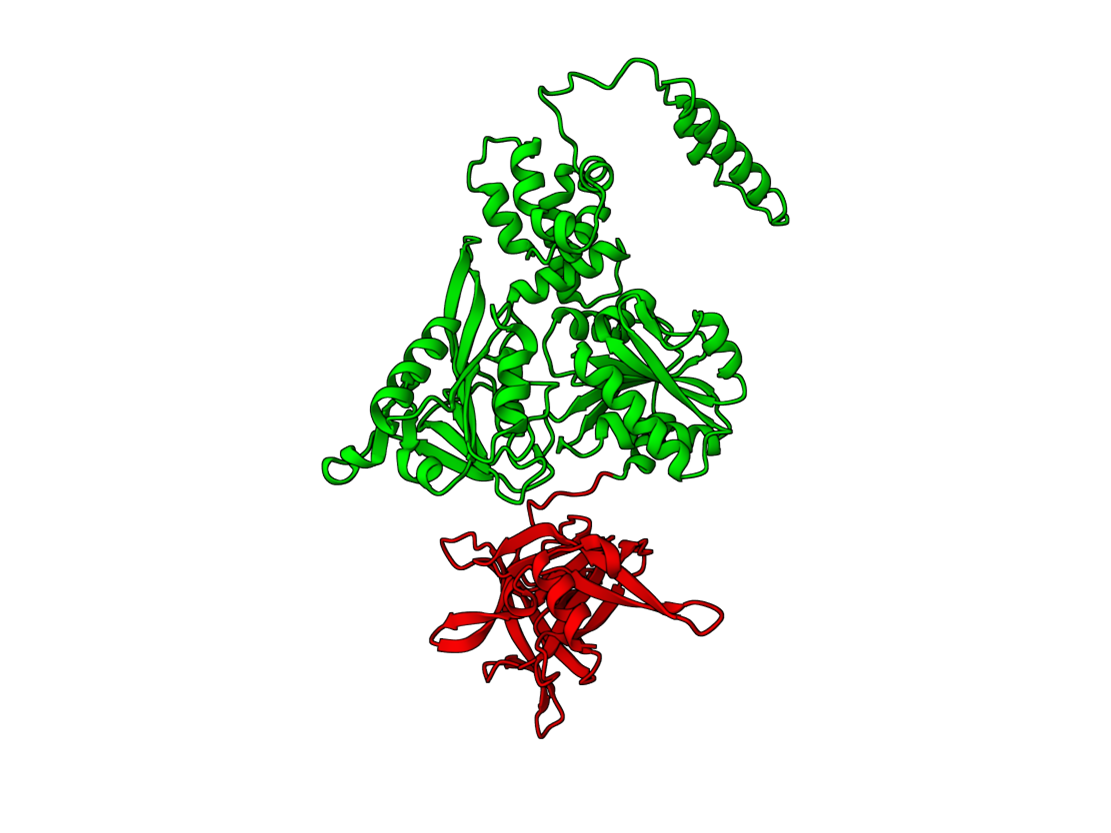

Supplement: Supplementary file 1 [file ijms-25-13654-s001.zip › Figure S3.TIF]

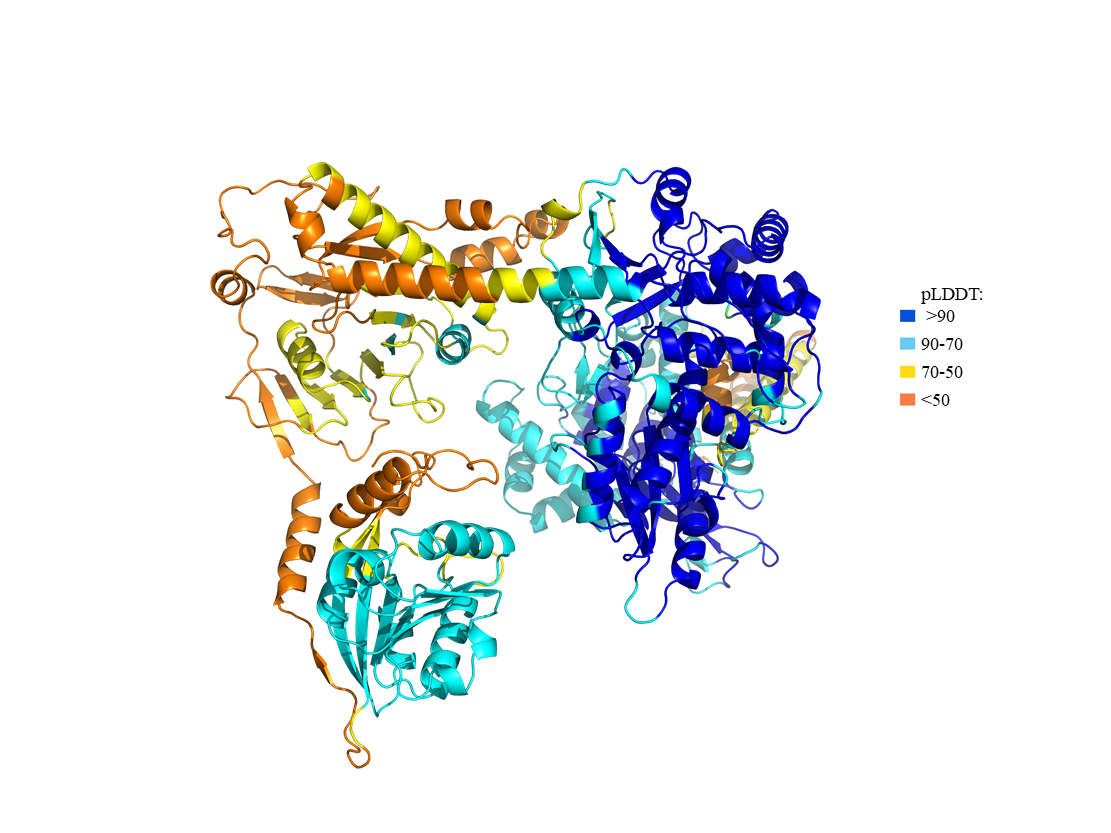

Supplement: Supplementary file 1 [file ijms-25-13654-s001.zip › Figure S30.TIF]

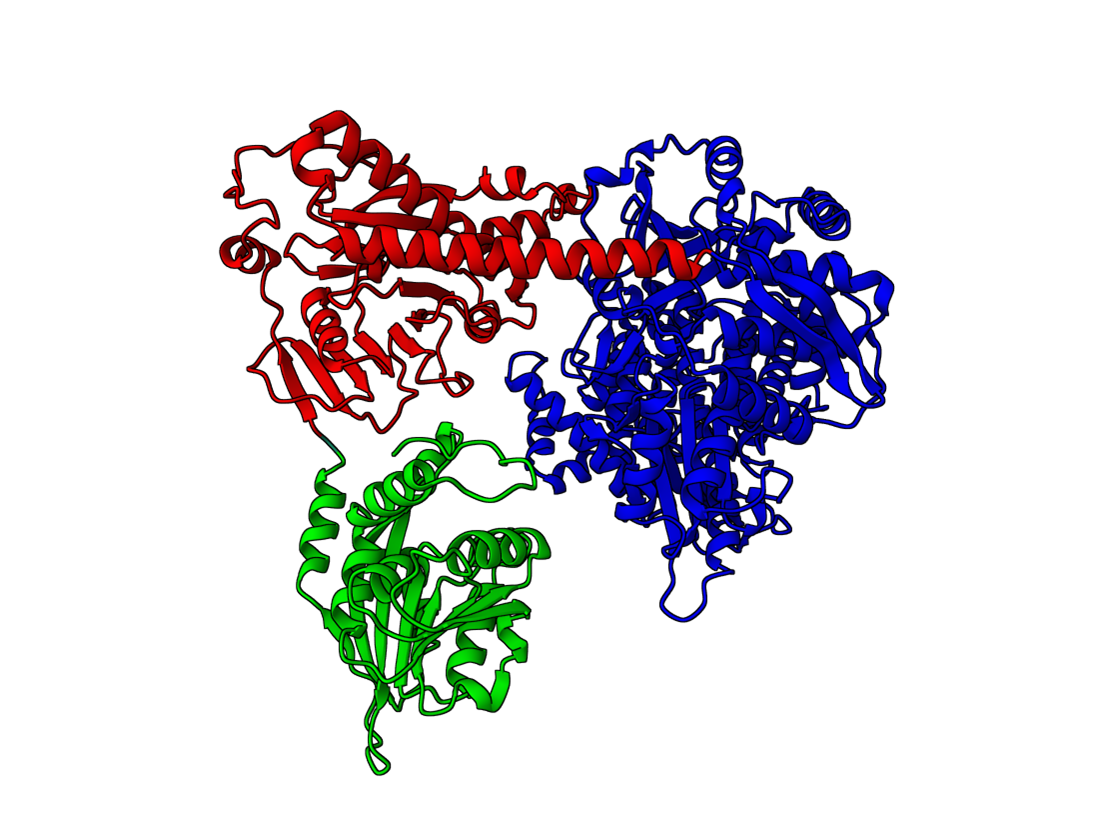

Supplement: Supplementary file 1 [file ijms-25-13654-s001.zip › Figure S31.TIF]

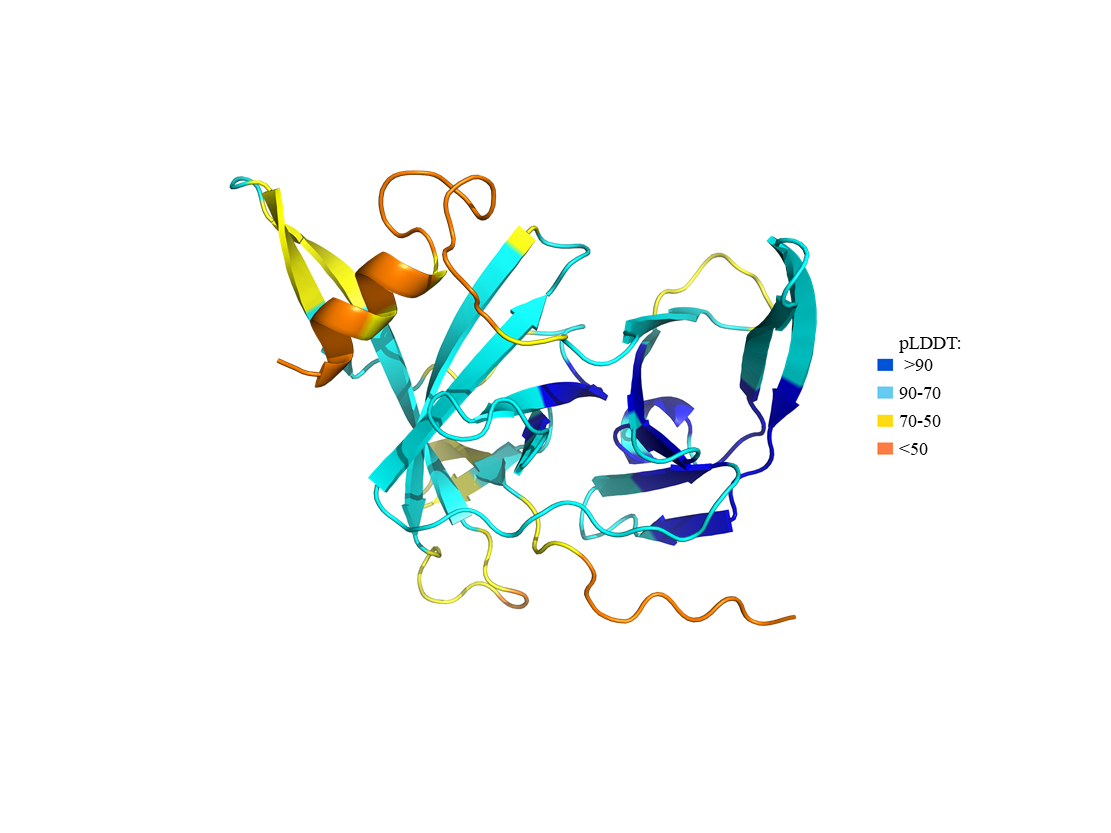

Supplement: Supplementary file 1 [file ijms-25-13654-s001.zip › Figure S4.TIF]

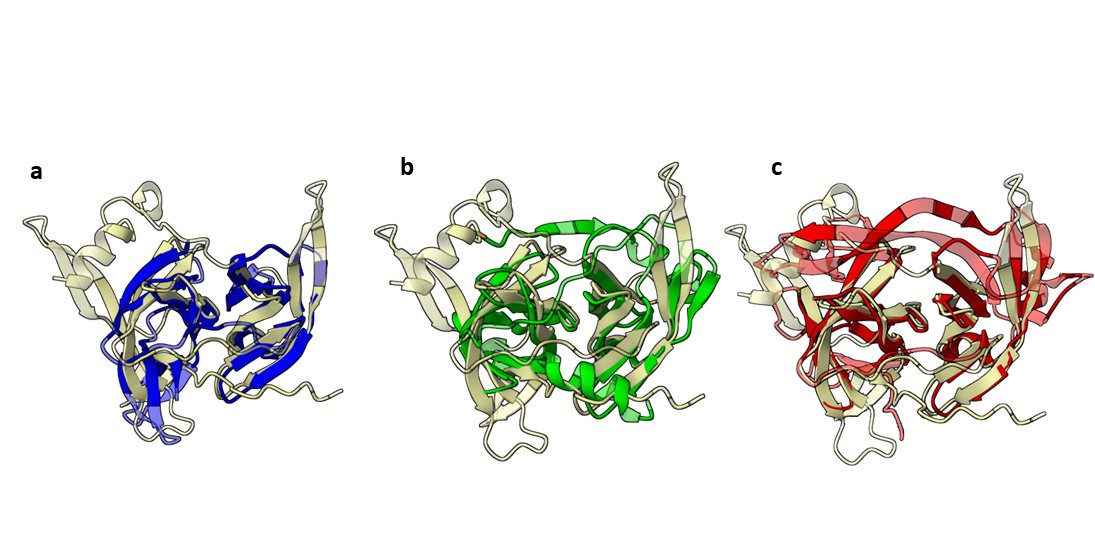

Supplement: Supplementary file 1 [file ijms-25-13654-s001.zip › Figure S5.TIF]

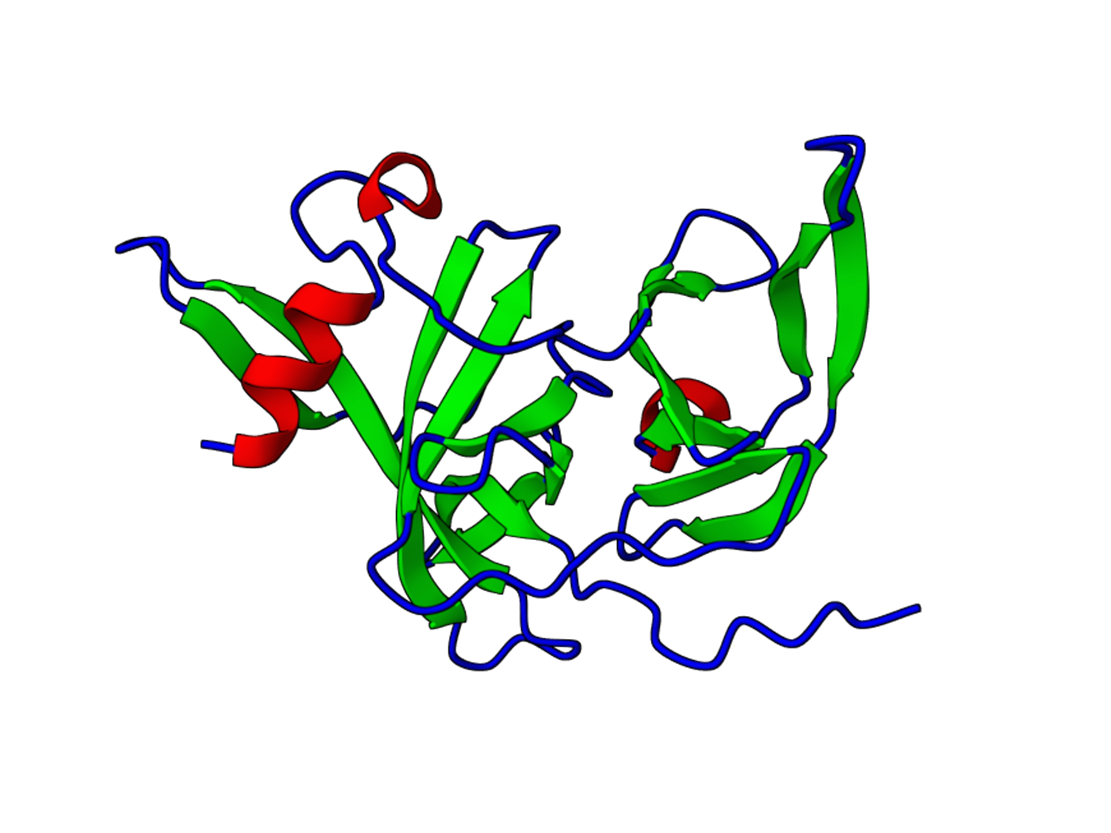

Supplement: Supplementary file 1 [file ijms-25-13654-s001.zip › Figure S6.TIF]

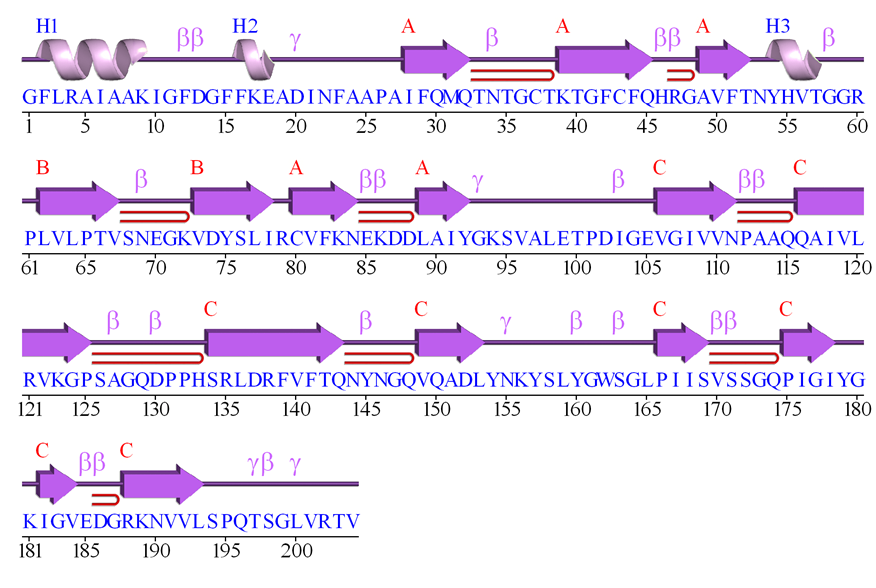

Supplement: Supplementary file 1 [file ijms-25-13654-s001.zip › Figure S7.TIF]

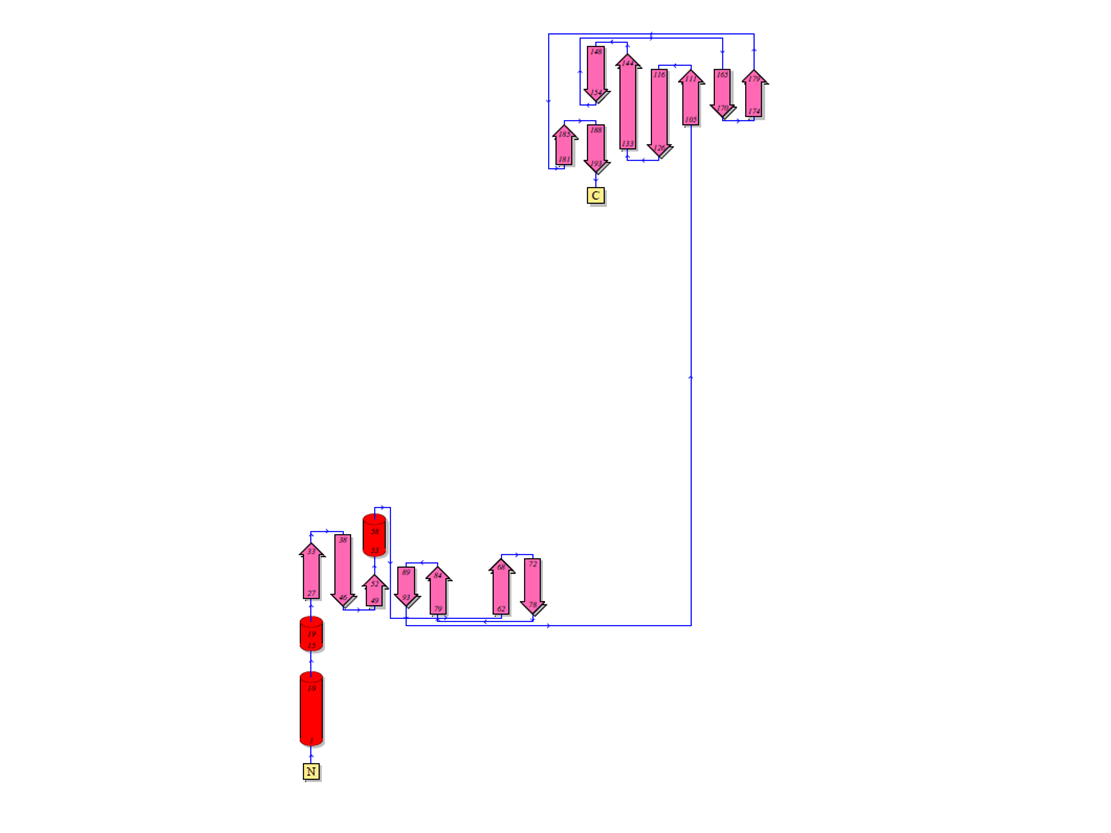

Supplement: Supplementary file 1 [file ijms-25-13654-s001.zip › Figure S8.TIF]

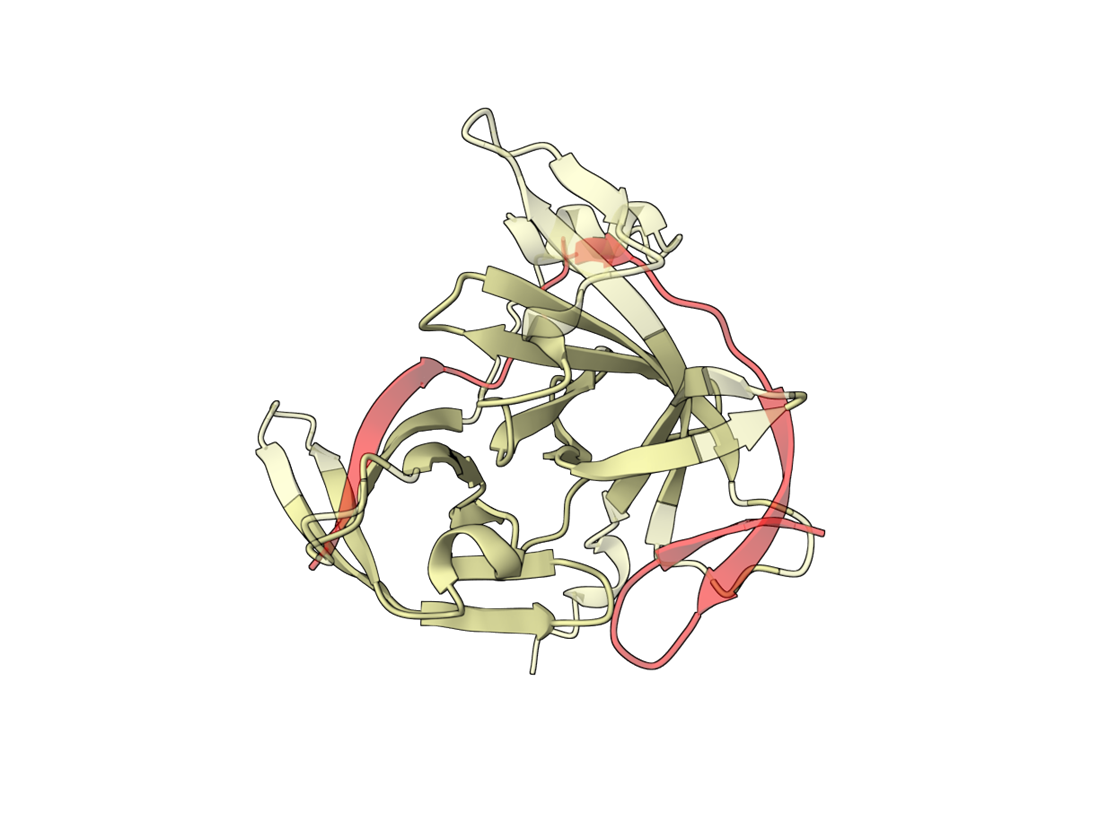

Supplement: Supplementary file 1 [file ijms-25-13654-s001.zip › Figure S9.TIF]
